# Canalization of Gene Expression and Domain Shifts in the *Drosophila* Blastoderm by Dynamical Attractors

**DOI:** 10.1371/journal.pcbi.1000303

**Published:** 2009-03-13

**Authors:** Manu, Svetlana Surkova, Alexander V. Spirov, Vitaly V. Gursky, Hilde Janssens, Ah-Ram Kim, Ovidiu Radulescu, Carlos E. Vanario-Alonso, David H. Sharp, Maria Samsonova, John Reinitz

**Affiliations:** 1 Department of Applied Mathematics and Statistics, and Center for Developmental Genetics, Stony Brook University, Stony Brook, New York, United States of America; 2 Department of Computational Biology, Center for Advanced Studies, St. Petersburg State Polytechnical University, St. Petersburg, Russia; 3 Theoretical Department, The Ioffe Physico-Technical Institute of the Russian Academy of Sciences, St. Petersburg, Russia; 4 EMBL/CRG Research Unit in Systems Biology, CRG – Centre de Regulació Genòmica, Barcelona, Spain; 5 Institute of Mathematical Research of Rennes, University of Rennes, Rennes, France; 6 Theoretical Division, Los Alamos National Laboratory, Los Alamos, New Mexico, United States of America; Princeton University, United States of America

## Abstract

The variation in the expression patterns of the gap genes in the blastoderm of the fruit fly *Drosophila melanogaster* reduces over time as a result of cross regulation between these genes, a fact that we have demonstrated in an accompanying article in *PLoS Biology* (see Manu et al., doi:10.1371/journal.pbio.1000049). This biologically essential process is an example of the phenomenon known as canalization. It has been suggested that the developmental trajectory of a wild-type organism is inherently stable, and that canalization is a manifestation of this property. Although the role of gap genes in the canalization process was established by correctly predicting the response of the system to particular perturbations, the stability of the developmental trajectory remains to be investigated. For many years, it has been speculated that stability against perturbations during development can be described by dynamical systems having attracting sets that drive reductions of volume in phase space. In this paper, we show that both the reduction in variability of gap gene expression as well as shifts in the position of posterior gap gene domains are the result of the actions of attractors in the gap gene dynamical system. Two biologically distinct dynamical regions exist in the early embryo, separated by a bifurcation at 53% egg length. In the anterior region, reduction in variation occurs because of stability induced by point attractors, while in the posterior, the stability of the developmental trajectory arises from a one-dimensional attracting manifold. This manifold also controls a previously characterized anterior shift of posterior region gap domains. Our analysis shows that the complex phenomena of canalization and pattern formation in the *Drosophila* blastoderm can be understood in terms of the qualitative features of the dynamical system. The result confirms the idea that attractors are important for developmental stability and shows a richer variety of dynamical attractors in developmental systems than has been previously recognized.

## Author Summary

C. H. Waddington predicted in 1942 that networks of chemical reactions in embryos can counteract the effects of variable developmental conditions to produce reliable outcomes. The experimental signature of this process, called “canalization,” is the reduction of the variation of the concentrations of molecular determinants between individuals over time. Recently, Waddington's prediction was confirmed in embryos of the fruit fly *Drosophila* by observing the expression of a network of genes involved in generating the basic segmented body plan of this animal. Nevertheless, the details of how interactions within this genetic network reduced variation were still not understood. We use an accurate mathematical model of a part of this genetic network to demonstrate how canalization comes about. Our results show that coupled chemical reactions having multiple steady states, or attractors, can account for the reduction of variation in development. The variation reduction process can be driven not only by chemical steady states, but also by special pathways of motion through chemical concentration space to which neighboring pathways converge. These results constitute a precise mathematical characterization of a healing process in the fruit fly embryo.

## Introduction

Canalization refers to the constancy of the wild type phenotype under varying developmental conditions [1]–[4]. In order to explain canalization, C. H. Waddington hypothesized that there must only be a finite number of distinct developmental trajectories possible, since cells make discrete fate decisions, and that each such trajectory, called a *chreod*, must be stable against small perturbations [5]. One aspect of canalization, the buffering of phenotypic variability against genotypic variability in wild type, has received considerable experimental [2], [6]–[10] and theoretical [11]–[13] attention. The phenomenon of canalization of genotypic and environmental variation was seen by Waddington as a consequence of the underlying stability of developmental trajectories, an idea supported by theoretical analysis [13]. But this central idea of Waddington's has heretofore received little attention in real developmental systems because of a lack of relevant quantitative molecular data. The further investigation of Waddington's hypothesis is of great importance because it provides a scientific connection between the reliability and invariance of the formation of cell types and tissues in the face of underlying molecular variability, as we now explain.

**Figure 1:**
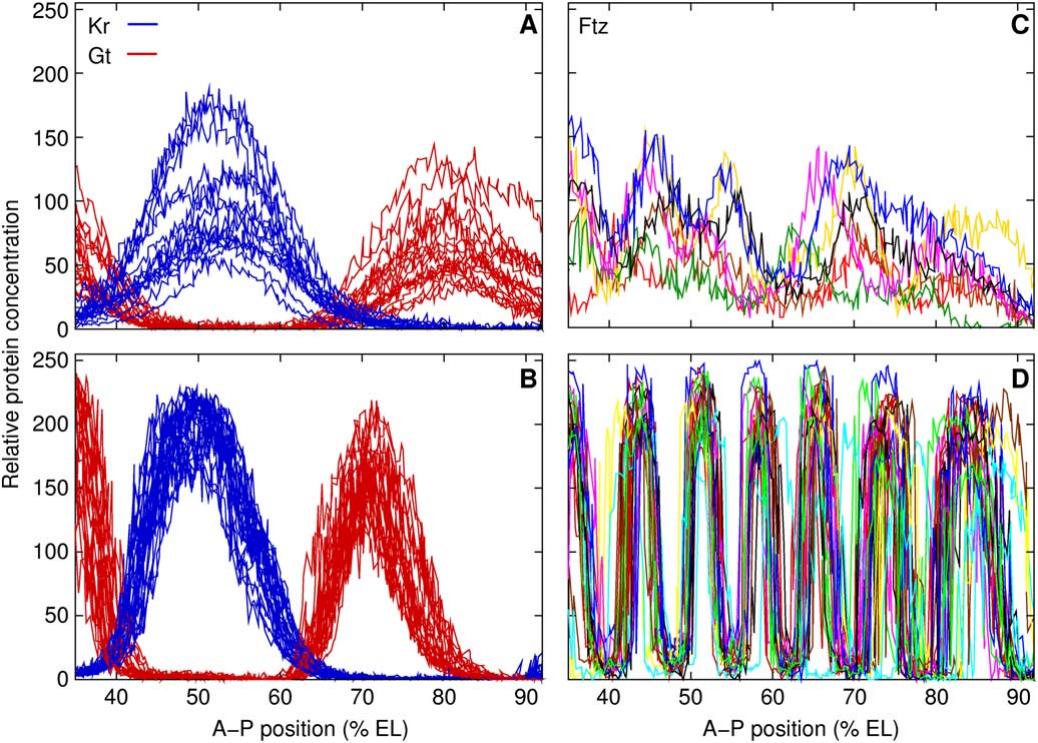
Reduction of variation in segmentation gene expression patterns over time. *Kr* and *gt* expression patterns 47 min (A, early) and 3 min (B, late) before gastrulation. *ftz* expression patterns 34 min (C, early) and 3 min (D, late) before gastrulation. The expression patterns shown here and subsequently are from the middle 10% of dorsoventral positional values; 0% egg length (EL) is the anterior pole. The standard deviation (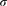) of expression level at the peak of the central *Kr* domain is 33.0 early (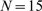 embryos), and reduces to 17.4 late (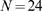). The position of the domain peak has 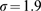 early and 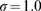 late. The expression level at the peak of the *gt* posterior domain has 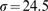 early (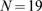), and 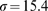 late (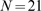). The position of the domain peak has 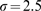 early and 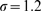 late. The expression pattern of *ftz* has extensive qualitative variation early (panel C, 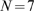), but is very reproducible just before gastrulation (panel D, 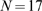). Embryos with the most diverse patterns of *ftz* were chosen for panel C, while they were randomly chosen for all other panels.

Quantitative molecular data permitting the study of developmental canalization are now available for the segment determination process in *Drosophila* [14]. The segmented body plan of the fruit fly *Drosophila melanogaster* is determined when the embryo is a blastoderm [15] by the segmentation genes [16]. Quantitative spatiotemporal gene expression data show that the maternal protein gradients and the early expression patterns of the zygotic gap and pair-rule genes vary a great deal from embryo to embryo [14],[17]. The variation of the expression patterns of the gap and pair-rule genes decreases over time so that it is significantly lowered by the onset of gastrulation at the end of cellularization ([14], Fig. 1). The observed reduction of variability over time in the segmentation gene system suggests that the developmental trajectory of the *Drosophila* embryo is stable against perturbation. The characterization of the stability properties of the developmental trajectory is central to our understanding of the mechanisms that underlie canalization [3].

In the case of the gap genes, we have shown elsewhere [18] that variation reduction relative to the maternal gradient Bicoid (Bcd) occurs because of gap gene cross regulation. Using a gene circuit model of the gap gene network [18]–[22] we identified specific regulatory interactions responsible for variation reduction *in silico* and verified their role in canalization experimentally. Importantly, the model reproduces the observed low variation of gap gene expression patterns [18], which provides an opportunity to analyze the properties of the system that give rise to developmental stability.

These results raise two generic problems that occur in the analysis of complex numerical models. First, even if the model describes a natural phenomenon faithfully, understanding the natural phenomenon is only achieved when the model's behavior can be understood as well. The complexity of the model, unsurprising in terms of the underlying complexity of the biological system itself, poses a significant challenge to understanding model function. Second, any model is an approximation to the actual mechanisms operating in an organism. The model's behavior must be robust to perturbation, since organisms develop and function reliably even though the underlying mechanisms are subject to a wide variety of perturbations and stresses. There is extensive molecular variability among cells and embryos ([14], [17], [23]–[26]; reviewed in [27]) and yet there is functional identity between equivalent cell types or conspecific individuals.

René Thom tried to resolve this apparent contradiction between the constancy of biological function and the variability in biological substructure by proposing a qualitative topological view of the trajectories of dynamical models [28]. The term “topology” is used here to refer to properties of developmental trajectories that are invariant under continuous deformation. The preservation of these properties ensures the robustness of model behavior, while a qualitative view often leads to an intuitive understanding of complex mechanisms. One such robust property is an attractor state, or a stable steady state of a dynamical system, that attracts all trajectories in some neighborhood of itself. Attractor states are locally stable under small perturbations of the dynamical model [29], and for this reason it has been proposed that cell fates are attractors [11], [13], [30]–[33].

The presence of an attractor state in the phase space of a system implies that there exists a region of phase space, called the basin of attraction, in which all trajectories approach the attractor asymptotically [34],[35]. This suggests that an attractor is the kind of qualitative robust property that could explain the stability of trajectories, and hence canalization. There are, however, three important considerations to keep in mind when using attractors to describe the *Drosophila* blastoderm. First, the reduction of variation due to attractors is only guaranteed at late times, but the reduction in the variation of the gap gene expression patterns takes place over about 100 minutes prior to gastrulation. The reduction of variation before gastrulation is biologically essential as the expression patterns of *engrailed* and *wingless*, which form the segmentation prepattern, have a resolution of one nucleus and are created by the precise overlap of pair-rule and gap domains [14],[36]. Furthermore, at about the time of gastrulation the embryo undergoes the midblastula transition [37],[38] at which time a qualitative change occurs in the genetic control of the embryo. Second, in general there can be more than one attractor in the phase space [39]–[43]. Thus, the basins of attraction need to correspond to biological initial conditions and be large enough to ensure robustness. Finally, the set of attractors found must succeed not only in explaining canalization but also the morphogenetic properties of the system. One such property is the anterior shift of gap gene domains located in the posterior region [14],[21],[44]. These shifts are biologically significant and are difficult to reconcile with stable point attractors.

In this paper we show that the variation reduction of gap gene expression patterns is a consequence of the action of robust attracting states. We further show that the complex patterning of the gap gene system reduces to the three qualitative dynamical mechanisms of (1) movement of attractors, (2) selection of attractors, and (3) selection of states on a one dimensional manifold. The last of the three mechanisms also causes the domain shifts of the gap genes, providing a simple geometric explanation of a transient phenomenon.

In the Gap Gene Circuits section we briefly describe the gene circuit model; see [18] for a full description. For each nucleus in the modeled anteroposterior (A–P) region, we identified the attractors in the gap gene phase space, calculated the trajectories, the basins of attraction and other invariant sets such as one dimensional attracting manifolds (Stability Analysis of the Trajectories of the Gap Gene System section). The stability of the trajectories was tested by varying the initial conditions within a biological range, based on gene expression data, that represents the variability of early gap gene expression. We plotted the attractors and several trajectories corresponding to different initial conditions to make phase portraits that show the global qualitative behavior of the system. Finally, we studied how the phase portraits changed as A–P position was varied to infer qualitative pattern formation mechanisms. The biological conclusions about canalization and pattern formation arising from the dynamical characterization are presented in the Mechanisms of Canalization and Pattern Formation section.

## Results

### Gap Gene Circuits

The gene circuit used in this study models the spatiotemporal dynamics of the protein expression of the gap genes *hunchback* (*hb*), *Krüppel* (*Kr*), *giant* (*gt*), and *knirps* (*kni*) during the last two cleavage cycles (13 and 14A) before gastrulation [37] in the *Drosophila* blastoderm. The protein products of these genes localize to nuclei [45]–[48] so that the state variables are the concentrations of the proteins in a one dimensional row of nuclei along the A–P axis of the blastoderm. The concentration of the 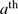 protein in the 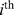 nucleus at time 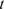 is denoted by 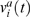. In the model we considered a region, from 35% to 92% egg lenth (EL) along the A–P axis, which corresponds approximately to the region of the blastoderm fated to form the segmented part of the adult body [49],[50].

**Figure 2:**
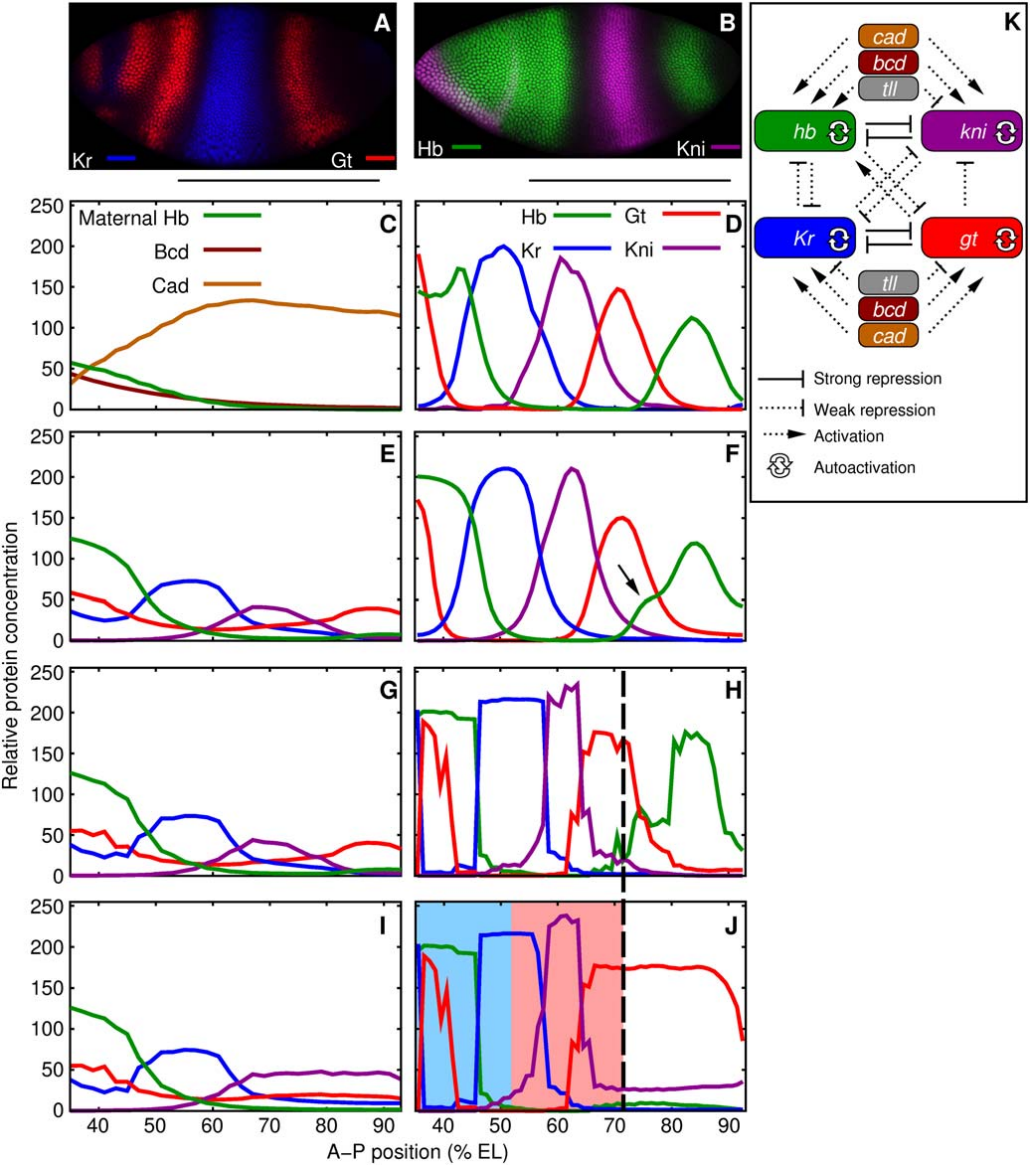
Gap gene data and expression patterns in the gene circuit chosen for analysis. (A,B) Time class T8 (∼3 mins before gastrulation; Table S1) embryos immunostained for Kr and Gt (A) and Hb and Kni (B). Anterior is to the left and dorsal is above. Bars indicate the modeled region. (C) Data for the maternal protein gradients Bcd (cleavage cycle 13), Hb (cycle 12), and Cad (cycle 12). (D) Average gap gene data in time class T8. (E,F) Cleavage cycle 13 (E) and time class T8 (F) gap gene expression patterns produced by the gene circuit. The arrow shows the main patterning defect, which is related to experimental noise in Tll data [18]. (G,H) Gap gene expression patterns produced by the same circuit in cleavage cycle C13 (G) and time class T8 (H) in the absence of diffusion (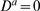 for all proteins). (I,J) Expression patterns produced by the circuit in cleavage cycle 13 (I) and time class T8 (J) in the absence of diffusion and *tll*. The dashed vertical line shows the region (35%–71% EL) in which the expression patterns of the circuit excluding *tll* (J) agree with the circuit that has *tll* (H). The anterior and posterior regions identified in the stability analysis (Mechanisms of Canalization and Pattern Formation section) are highlighted in panel J in blue and red respectively. (K) The topology of the gap gene network determined by the gene circuit method.

The gap genes are expressed in broad domains (Fig. 2A,B; [14]) under the control of maternal cues. The anterior maternal system acts primarily through the protein gradient Bcd [51]–[53] which is essentially stationary and has an exponential profile (Fig. 2C; [14],[51],[54]) during the modeled time period. The posterior maternal system is represented by the maternal Hb gradient (Fig. 2C; [55]–[57]). The terminal system regulates gap gene expression by activating *tailless* (*tll*) and *huckebein* (*hkb*) [58]–[61]. The terminal system is represented in the model by the Tll gradient, which is expressed posterior to 80% EL in the modeled region during cycles 13 and 14 ([14] and Fig. S1B). *tll* is considered upstream of the gap genes since its expression pattern is unchanged in gap gene mutants [62]. The concentration of Bcd in nucleus 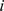 is denoted by 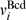 and was determined using Bcd data from a representative cycle 13 embryo by an exponential fit, so that 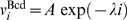 (see [18] for details). The concentrations of Tll and another upstream regulator, Caudal (Cad) [63],[64], were determined by interpolating average data in time [18]. The concentrations of Tll and Cad are denoted by 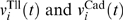 respectively, with an explicit dependence on time, since these gradients are not stationary (Fig. S1).

The dynamical equations governing 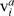 are given by
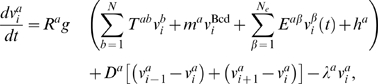
(1)where 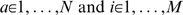 in a gene circuit with 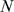 genes and 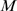 nuclei. The first term on the right hand side of Eq. (1) represents protein synthesis, the second one represents protein transport through Fickian diffusion and the last term represents first-order protein degradation. The diffusion constant, 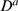 varies inversely with squared internuclear distance, and 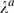 is the degradation rate. The synthesis term is set to zero during the mitosis preceding the thirteenth nuclear division as synthesis shuts down [65]. Following this mitosis, the nuclei are divided and daughter nuclei are given the same state as the mother nucleus.


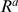
 is the maximum synthesis rate, and 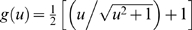 is a sigmoidal regulation-expression function. The first term in the argument of 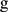 represents the transcriptional cross regulation between the gap genes and the genetic interconnectivity is specified by the matrix 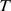. Positive elements of 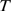 imply activation while negative ones imply repression. The regulation of the gap genes by Bcd is represented in the second term and 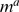 is the regulatory strength. The regulation of the gap genes by upstream time-varying inputs is represented in the third term and 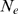 is the number of such inputs. There are two such inputs in this model, Cad and Tll, and the elements of the matrix 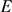 have the same meaning as those of 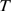. The last term, 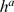, represents the effect of ubiquitous transcription factors and sets the threshold of activation.

The initial conditions for Hb are specified using cleavage cycle 12 data. Cycle 12 data are a good approximation to the maternal Hb gradient since the zygotic expression of *hb* appears to begin in cleavage cycle 13 [17]. The initial conditions for Kr, Gt, and Kni are taken to be zero, since their protein expression is first detected in cycle 13 [14], [61], [66]–[68].

The gene circuit's parameters were determined by performing a least-squares fit to a time series of averaged gap gene data [14] using the Parallel Lam Simulated Annealing algorithm (see Methods). This time series has nine points (time classes; see Table S1), one in cycle 13 and the rest in cycle 14A. The output of the gene circuit (Fig. 2E,F) fits the data (Fig. 2D) well and its network topology (Fig. 2K) is consistent with previous results (see [18] for discussion and parameters).

### Stability Analysis of the Trajectories of the Gap Gene System

In order to characterize the stability of the trajectories of the gap gene system in terms of qualitatively robust features like attractors, we apply the tools of dynamical systems theory [34],[69]. Since the gene circuit has 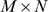 variables (Gap Gene Circuits section) its state is represented as a point in an 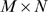-dimensional concentration space, or phase space. In general the concentrations of gap proteins change with time, and hence, a solution of the gene circuit is a curve in this phase space. The gene circuit can also have solutions which do not change with time. Such a solution, called an equilibrium or steady state solution, is represented as a single point in phase space. The positions of the equilibrium solutions in phase space and their stability properties determine the stability of a general time varying solution of the gene circuit. The reader not familiar with linear analysis near an equilibrium point should see Protocol S2 for a pedagogical description of equilibria and their stability in two dimensions.

#### Diffusionless approximation

In the gap gene circuit used in this study (Gap Gene Circuits section), there are 58 nuclei and 4 gap genes, giving rise to a dynamical system having 232 dimensions. Such a large number of dimensions pose a significant challenge to visualizing the results of the phase space analysis. In order to make the analysis tractable, we made the approximation that there is no diffusion, 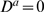 in Eq. (1), while keeping all other parameters in Eq. (1) at their original values [18]. This uncouples the nuclei and the system of 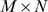 coupled ODEs reduces to a set of 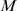 independent systems of 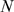 ODEs. Eq. (1) thus becomes
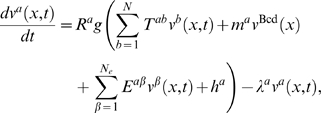
(2)where the dependence of the concentrations of gap gene proteins, Bcd and time varying external inputs on the location of nucleus 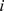 along the A–P axis is denoted by 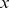.

In the absence of diffusion, the model still gives the correct sequence of gap gene domains (Fig. 2G,H); and the border positions are close to their values in the presence of diffusion (Table S2). In contrast to the circuit with diffusion however, the domains have sharp boundaries and have little or no overlap (Fig. 2H). The general agreement between the circuits with and without diffusion allows the analysis of the reduced system and supports the result from earlier work [21] that diffusion is not required for making gap gene expression patterns.

In Eq. (2), the terms 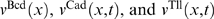 represent the anterior and terminal maternal systems. They parametrize the set of solutions possible in each nucleus as a function of A–P position. From the set of solutions specified by the concentrations of Bcd, Cad, and Tll, a particular solution is chosen by the initial conditions specified by the concentration of maternal Hb (Gap Gene Circuits section; Fig. 2C). Therefore the posterior maternal system specifies position in a manner distinct from the anterior and terminal systems. We further simplified the analysis by neglecting the effects of Tll in Eq. (2), giving
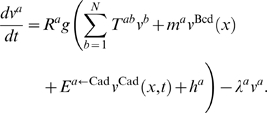
(3)


The gap gene expression patterns (Fig. 2I,J) produced by Eq. (3) agree with a model that includes *tll* (Eq. 2) until 71% EL, therefore the simplified analysis is valid in the region from 35% EL to 71% EL.

#### Equilibria, stability, and bifurcations

We considered each nucleus in cleavage cycle 13 and its posterior daughter in cycle 14A since the anterior daughter differs only slightly from the posterior one in the concentrations of Bcd and Cad. For simplicity we refer to mother-daughter pairs by the daughter nuclei, which are located at odd numbered positions from 35% to 71% EL. For each nucleus we determined the equilibria and, based on maternal Hb data (Fig. 2C), the family of solutions corresponding to the observed range of initial conditions. The concentration of Cad (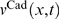) varies with time (Fig. S1A), implying that the equilibria in the phase space of a nucleus also change with time. We are interested in studying the properties of the solutions during late cycle 14A, since the variation in gap gene expression levels is least in that time period (Fig. 1). Also, the stationary approximation for Bcd is valid until time class T6 (∼16 mins before gastrulation; Table S1) as Bcd concentration decreases two-fold afterward [14]. For these reasons, we calculated equilibria at time class T6 (Protocol S1: Eq. S3). The trajectories were calculated using time varying Cad data until time class T6 and with T6 Cad data thereafter (Protocol S1: Eq. S1) so that their late time behavior corresponded to the calculated equilibria.

The equilibria were calculated using the Newton-Raphson method [70],[71] and classified according to their stability (Protocol S3). We calculated several trajectories with different initial conditions to test variation reduction and stability, and continued the integration of Eq. (S1) (Protocol S1) to very late times in order to visualize asymptotic behavior. Since the segments are determined by the time of gastrulation, significant variation reduction must occur beforehand for the precise specification of position. Hence, we distinguish the biological behavior from asymptotic behavior graphically. We found that each nucleus has multiple attractors and that its trajectory can potentially approach any one depending on the initial condition. It was necessary, therefore, to characterize the basins of attraction [34],[69] of the attractors. We exploited the fact that only Hb has non-zero initial conditions (Gap Gene Circuits section) to characterize the basins as intervals on the Hb axis (see Protocol S3 for details of calculation). We also calculated one dimensional unstable manifolds [72] of saddle points to better understand the transient behavior of solutions (Protocol S3).

**Figure 3:**
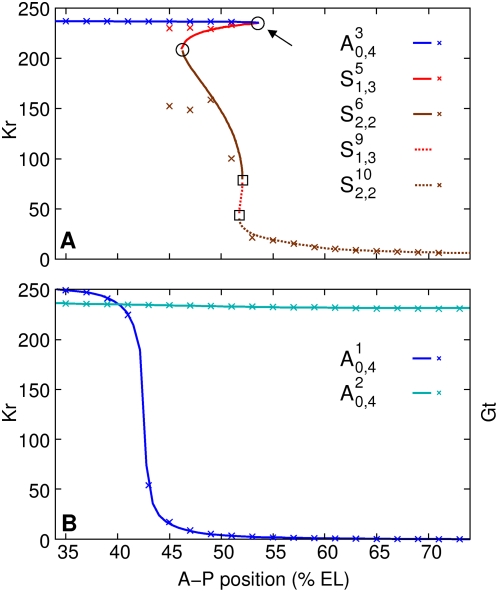
Equilibria determined by the continuous analysis. The points are equilibria calculated by Newton-Raphson at discrete nuclear positions using Cad data (red curve in Fig. S3), while the solid lines are equilibria determined by the continuous analysis using the interpolated Cad profile (black curve in Fig. S3). Point attractors are blue or cyan and saddle equilibria having one or two eigenvalues with positive real part are red or brown respectively. The 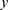-axis is the projection of equilibria positions on the Kr axis (A and B) or the Gt axis (B). The 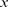-axis is the bifurcation parameter, the A–P position 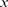. (A) The bifurcations observed in both the analysis at discrete positions and the continuous analysis are encircled. The bifurcations only seen in the continuous analysis are highlighted in boxes. The arrow highlights the annihilation between 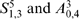 at 53% EL that divides the anterior and posterior regions. See Table S4 for bifurcation values of the A–P position. (B) The point attractor 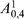 (right 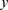-axis), showing its continuous movement from the *hb,gt*-on to the *hb*-on state. 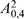 is plotted on the left 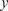-axis.

The analysis of the phase space for different nuclei revealed several A–P positions at which the set of equilibria changes. This suggests that the dynamical system is undergoing bifurcations resulting from the changing values of its parameters, the concentrations of Bcd and Cad, as A–P position is varied. Since we used Cad data in Eq. (3), it is also possible that these bifurcations are artifacts caused by fluctuations in the data. We verified that the changes observed in the analysis at discrete odd numbered positions were actual bifurcations by varying the concentrations of Bcd and Cad, parametrized by the A–P position, continuously (Protocol S4). This was done by interpolating the Cad data with a cubic polynomial (Fig. S3). Note that the Bcd concentration profile has already been parametrized as a function of position (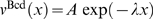; Gap Gene Circuits section), and can be varied continuously. The continuous analysis validated all the bifurcations at discrete locations (Fig. 3A and Fig. S4; circles) save for two. These two bifurcations (Fig. 3A; boxes) could not be observed in the analysis at discrete locations as they occur consecutively between two nuclei and leave the set of equilibria unchanged. Although the bifurcations occur at particular values of the concentrations of Bcd and Cad, subsequently we will refer to them by A–P position for simplicity.

We further characterized the type of bifurcations (Protocol S4). Though many types of bifurcations are possible only one type, the saddle-node (see Fig. S2 for illustration in two dimensions), occurs in the gap gene system. In the four-dimensional gap gene system any creation or annihilation of a pair of equilibria that differ in the sign of one eigenvalue is a saddle-node bifurcation (Fig. 3A and Fig. S4).

### Mechanisms of Canalization and Pattern Formation

Based on the analysis in the previous section the region of interest, from 35% EL to 71% EL, can be divided into an anterior and a posterior region (Fig. 2J) having distinct modes of canalization and pattern formation. The two regions are separated by a saddle-node bifurcation that occurs at 53% EL (Fig. 3A), that is, at the peak of the central *Kr* domain.

We next demonstrate that in the anterior region (Fig. 2J), which extends from the peak of the third anterior *gt* domain to the peak of the central *Kr* domain, the state of a nucleus at gastrulation is close to a point attractor. The trajectories are stable by virtue of being in the basin of attraction of the nucleus's attractor state and hence canalize. Pattern formation occurs by the selection of one state from many in a multistable phase space. The concentrations of the Bcd and Cad gradients control pattern formation in the anterior by determining the sizes of the basins and the positions of the attractors, while maternal Hb concentration selects a particular attractor by setting the starting point in its basin. Previous experimental [73] and theoretical [19] work suggested that Bcd and maternal Hb patterned the anterior of the embryo synergistically; our results identify specific roles for Bcd and Hb in anterior patterning.

The posterior region extends from the peak of the central *Kr* domain to the peak of the posterior *gt* domain (Fig. 2J) and its nuclei have phase spaces with very different properties. In this region, the state of the nucleus is far from any attractor state at gastrulation. Instead the state of a nucleus is close to a one-dimensional manifold and canalization is achieved due to attraction by this manifold. Even though the phase space is multistable, the biological range of maternal Hb concentrations in the posterior region place all nuclear trajectories in one basin of attraction. As a consequence, the modes of pattern formation operative in the anterior cannot function in the posterior. Maternal Hb patterns the posterior by determining the position on the attracting manifold which a particular trajectory reaches by the time of gastrulation. These results reveal the mechanism by which maternal Hb acts as a morphogen in the posterior [73]–[75] and also explain the dynamical shifts of gap gene domains [14],[21], a significant biological property of the posterior region.

We begin the presentation of detailed results by describing the phase spaces of typical nuclei in the two regions, highlighting mechanisms for canalization and pattern formation. An equilibrium is labeled by either 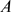 (point attractor) or 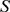 (saddle equilibrium), denoted by a superscript, with subscripts denoting the number of eigenvalues having positive or negative real parts. For example 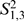 denotes the second saddle equilibrium in the modeled region which has one eigenvalue with positive real part and three with negative real parts. Equilibria are also given descriptive names based on which proteins are at high levels (*on*) ignoring the proteins that are at low levels. For example, if a point attractor is at *hb*-on, *Kr*-off, *gt*-on, and *kni*-off, it is referred to as the “*hb,gt*-on” attractor.

#### The anterior region

**Figure 4:**
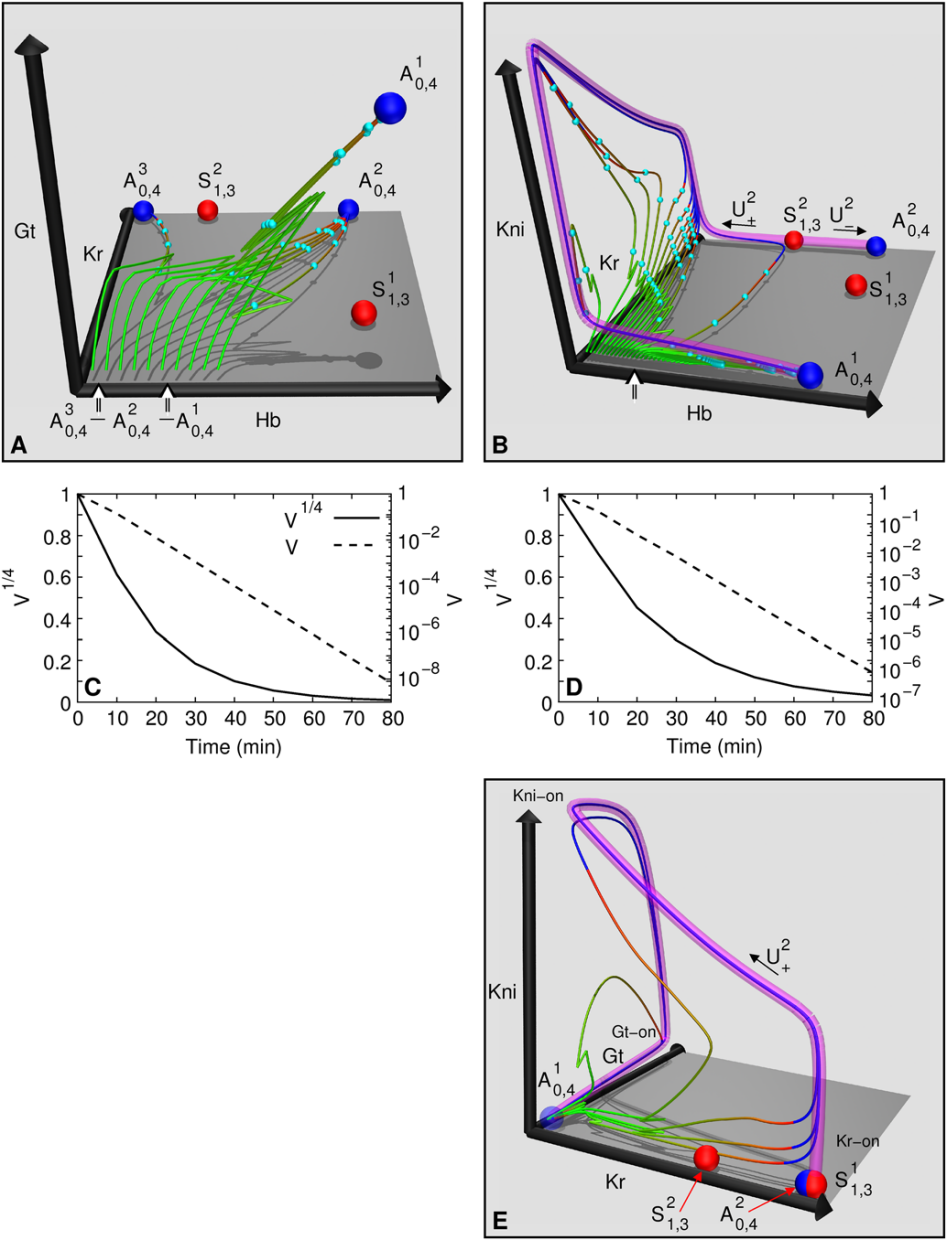
Two distinct dynamical regimes that control canalization. (A,B) Three-dimensional projections of four-dimensional phase portraits. Point attractors are blue and saddle equilibria having one eigenvalue with positive real part are red. Time is represented as a color gradient along the trajectories, with start of cycle 13 as green, and gastrulation as red; trajectories are blue after gastrulation. The midpoints of time classes (Table S1) T1, T3, T5, and T7 are indicated with cyan points. The sharp bends in trajectories are mitoses, when synthesis shuts down. See caption of Fig. S5 for details of rendering the phase portraits. (A) Hb-Kr-Gt projection of the phase portrait at 37% EL, highlighting the anterior dynamical regime. 10 trajectories are plotted. (B) Hb-Kr-Kni projection of the phase portrait at 57% EL, highlighting the posterior dynamical regime. 25 trajectories are shown. (C,D) Reduction of initial variation. (C) The time evolution of the volume of the box (52,100)×(0,1)×(0,60)×(0,1) representing initial variation in the anterior region nucleus at 37% EL. Dashed line is volume 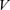 in log-scale; it reduces by a factor ∼10^8^ by gastrulation. 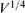 (solid line) gives the average shrinkage in a dimension. By gastrulation, each dimension shrinks by a factor of ∼20. 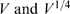 are shown normalized to 1. (D) The time evolution of the volume of the box (0,20)×(0,80)×(0,80)×(0,80) representing initial variation in the posterior region nucleus at 57% EL. 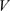 reduces by a factor of ∼10^6^ by gastrulation, and each dimension shrinks by an average factor of ∼10. (E) Kr-Gt-Kni projection of the phase portrait at 57% EL showing that the manifold 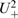 traverses the gap gene states in the posterior region, *Kr*-on, *kni*-on, and *gt*-on.

Fig. 4A shows the phase portrait of the nucleus at 37% EL from the anterior region. Since the phase space is four-dimensional, a three-dimensional projection onto the Hb-Kr-Gt axes is shown (see Fig. S6 for other projections). There are three attractors, 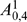 (*hb,gt*-on), 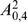 (*hb,Kr*-on), and 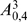 (*Kr*-on) shown in blue. Two saddle equilibria, 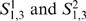, are shown in red. Saddle equilibria that are unimportant for the dynamics are not shown (see Protocol S5 and Table S4). In order to show the family of trajectories allowed by the values of Bcd and Cad concentrations in this nucleus, trajectories are plotted with starting points distributed uniformly on the Hb axis between 0–100, corresponding to the observed range of maternal Hb in the modeled region [14]. Time is represented as a color gradient along the trajectories, with the start of cycle 13 green, and gastrulation red. Trajectories are blue after gastrulation to indicate their asymptotic behavior.

The red-to-blue transition on the trajectories occurs very close to the attractors (Fig. 4A) implying that the nucleus is close to equilibrium at gastrulation. It is apparent by visual inspection that the initial variation of the trajectories is reduced dramatically by the onset of gastrulation, that is, trajectories are stable due to the point attractor. To verify this property numerically, we constructed a four-dimensional box such that its dimensions corresponded to the range of gap protein concentrations observed in cycle 13 and its volume represented initial variation. We then calculated the time evolution of the volume of this box using the model (Fig. 4C; see Protocol S8 for details of calculation). Variation in the concentration of each protein shrinks by a factor of ∼20 on average by gastrulation, confirming the stability of the developmental trajectory.

The concentration of maternal Hb, depending on which basin of attraction it lies in, selects a particular attractor state. The boundaries between the basins of attraction of the three attractors are indicated by arrows in Fig. 4A (see Protocol S5 for details). The concentration of maternal Hb in the nucleus at 37% EL puts it in the basin of the *hb,gt*-on (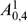) attractor (Table 1). This analysis correctly accounts for the observed gap gene expression in the nucleus, which lies at the peak of the third anterior *gt* domain and in the anterior *hb* domain (Fig. 2F,H). Posterior to this nucleus and up to 45% EL, all nuclei remain in the basin of the 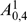 attractor (Table 1). 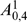 changes its position in the phase space from *hb,gt*-on at 37% EL to *hb*-on at 43% EL (Table 1 and Fig. 3B). The posterior border of the third anterior *gt* domain therefore forms by the movement of 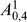 in the phase space. Since the Bcd and Cad concentrations are the only parameters being varied, the movement of an attractor is one mechanism by which Bcd patterns the anterior.

**Table 1:** Basins of point attractors in the anterior region and their selection by the value of maternal Hb concentration.

Nuc. (%EL)	Basin 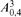	Basin 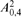	Basin 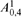	Mat. Hb conc.	Selected basin
35	NA	(0,58.20)	(58.20,100)	57.43	(*hb,Kr*-on) 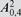
37	(0,2.05)	(2.05,52.12)	(52.12,100)	53.63	(*hb,gt*-on) 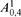
39	(0,10.61)	(10.61,47.87)	(47.87,100)	50.05	(*hb,gt*-on) 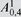
41	(0,19.01)	(19.01,42.98)	(42.98,100)	47.73	(*hb,gt*-on) 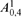
43	(0,26.29)	(26.29,41.04)	(41.04,100)	42.27	(*hb*-on) 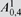
45	(0,30.79)	(30.79,37.46)	(37.46,100)	39.12	(*hb*-on) 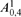
47	(0,34.18)	(34.18,37.76)	(37.76,100)	32.69	(*Kr*-on) 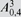
49	(0,35.24)	(35.24,36.91)	(36.91,100)	29.40	(*Kr*-on) 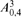
51	(0.94,38.31)	(38.31,39.26)	(39.26,100)	23.39	(*Kr*-on) 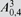

The basins of the attractors 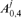 are shown as sets of initial (maternal) Hb concentrations in the second. third, and fourth columns. The value of the maternal Hb concentration in the circuit is shown in the fifth column. The last column shows the attractor in whose basin the initial condition lies.

The nucleus at 47% EL is in the basin of the *Kr*-on (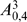) attractor, unlike the nuclei to its anterior, which are in the basin of *hb*-on (Table 1). The switch from *hb*-on to *Kr*-on causes the formation of the posterior border of the anterior *hb* domain and the anterior border of the central *Kr* domain between 45% EL and 47% EL (Fig. 2F,H). This happens due to 1) an increase in the size of the basin of the *Kr*-on attractor and 2) a decrease in the concentration of maternal Hb (second and fifth columns of Table 1). As is the case with the movement of the 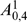 attractor, the first effect is due to the changing value of Bcd concentration. Hence, Bcd and maternal Hb pattern the anterior region in tandem [19],[73]; Bcd has the role of setting attractor positions and the extent of basins and maternal Hb selects a particular basin.

#### A bifurcation separating the anterior from the posterior

The nuclei remain in the basin of the *Kr*-on attractor up to 53% EL, where a saddle-node bifurcation annihilates *Kr*-on (Fig. 3A). Although there are other bifurcations in the A–P region considered (Table S3), only this bifurcation affects the dynamics of the gap gene system in a significant way. Posterior to this bifurcation the remaining two attractors *hb*-on (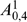) and *hb,Kr*-on (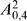) persist to the end of the region being analysed. No new attractors that might correspond to gap gene expression domains of the posterior appear, suggesting that the mechanisms of canalization and pattern formation are different from the anterior.

#### The posterior region

Fig. 4B shows the Hb-Kr-Kni projection (see Fig. S8 for others) of the phase portrait of the nucleus at 57% EL in the posterior region. There are two attractors 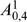 (*hb*-on) and 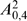 (*hb,Kr*-on). Two saddle equilibria, 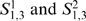, are shown, see Protocol S6, Fig. S7, and Table S4 for others. The basin of attraction of 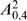 is very small ((43.99, 44.04)) and is shown with an arrow. The basin of attraction of 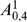 is divided into two intervals by the basin of 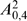. Trajectories right of the arrow (“direct interval”) reach close to 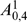 by gastrulation. Trajectories from the “indirect interval” to the left of the arrow are not close to the attractor at gastrulation as can be seen from the red-to-blue transitions. The direct trajectories are nonbiological since the concentration of maternal Hb (Fig. 2C) in the posterior region nuclei places all biological trajectories in the indirect interval.

Although the biological indirect trajectories are not in the vicinity of an attractor at gastrulation, they appear to be converging to a single trajectory in the phase space. This trajectory can be visualized by following the blue (post-gastrulation) segments of the trajectories in Fig. 4B. Therefore the trajectories are demonstrating stability, which we verified numerically by calculating the time evolution of a volume representing initial variation (Fig. 4D). The average variation in the concentration of each protein reduces by a factor of ∼10 by gastrulation. In order to understand this puzzling stability further, we calculated the one-dimensional unstable manifolds of the saddle equilibria 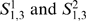. The unstable manifold of 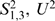, is shown as a translucent magenta tube in Fig. 4B. This manifold is precisely the trajectory to which all indirect trajectories are converging. Furthermore, the trajectories are close to this manifold by gastrulation (red-to-blue transitions). Hence this attracting manifold plays the same role in the posterior as attractors do in the anterior, and is responsible for the stability of trajectories and canalization (see also Protocol S7).

The attracting manifold 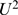 is also important for pattern formation in the posterior region. The positions and stability of equilibria in the phase space are qualitatively the same in all nuclei of the posterior region. This is a reflection of the relative constancy of Bcd, which is shallow due to exponential decay with position, and Cad, which has almost uniform expression in the posterior region (Fig. 2C; [14]). The invariance of the qualitative properties of the phase space implies that Bcd does not provide positional information in the posterior, a result arrived at by different means in other work [18],[21].

**Figure 5:**
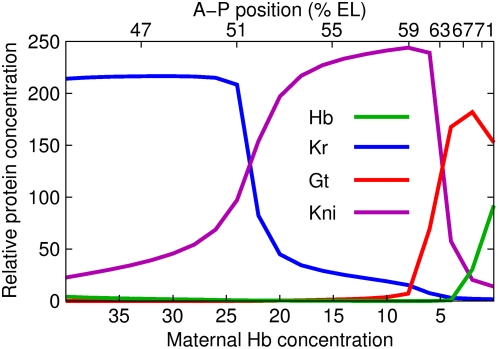
Maternal Hb is the morphogen in the posterior region. Gap gene concentrations at the midpoint of T8 in a single nucleus as a function of initial Hb concentration. The initial Hb concentration was varied uniformly in the nucleus at 63% EL (peak of the *kni* domain at gastrulation), keeping the Bcd and Cad inputs constant. The nucleus produces all gap gene states in the posterior region from the *Kr* peak (53% EL) to the *gt* peak (71% EL) as initial Hb concentration is decreased from 40 to 0. The shapes of the “domains” are distorted since maternal Hb has faster than linear decay with position; as a consequence anterior “domains” are exaggerated here. The 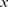-axis on the top shows A–P positions determined from the values of maternal Hb, showing that the domains are in correct proportions spatially. Posterior region nuclei form domains by responding to maternal Hb without any instruction from Bcd.

In order to understand how pattern formation occurs in the posterior, it is important to note two properties of the phase space (Fig. 4B). First, the attracting manifold 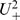 traverses all the posterior region states (*Kr*-on, *kni*-on, *gt*-on, and intermediate values; see Fig. 4E), before reaching 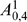. Second, the state achieved by an indirect trajectory at gastrulation has a continuous dependence on initial Hb value. High values of maternal Hb (20–40) in a nucleus lead to a *Kr*-on state, intermediate levels (12–20) lead to a *kni*-on state and lower values (4–8) lead to a *gt*-on state. Since maternal Hb decreases monotonically with A–P position (Fig. 2C), these two properties lead to the formation of gap gene expression patterns in the posterior region. In fact, changing only the maternal Hb concentration in a single posterior region nucleus (Bcd concentration is fixed), produces correct gap gene expression patterns in the posterior region (Fig. 5). This explains the experimental result that maternal Hb alone can pattern the embryo, if the anterior and terminal systems are absent, producing the posterior region expression patterns over the length of the whole embryo [75]. There is a further decrease in variability within the subintervals of maternal Hb concentration that lead to a particular gap gene state at gastrulation (see Protocol S7 and Fig. S9 for details). These results establish that maternal Hb is the morphogen in the posterior and patterns the region by selecting states on the attracting manifold 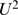.

**Figure 6:**
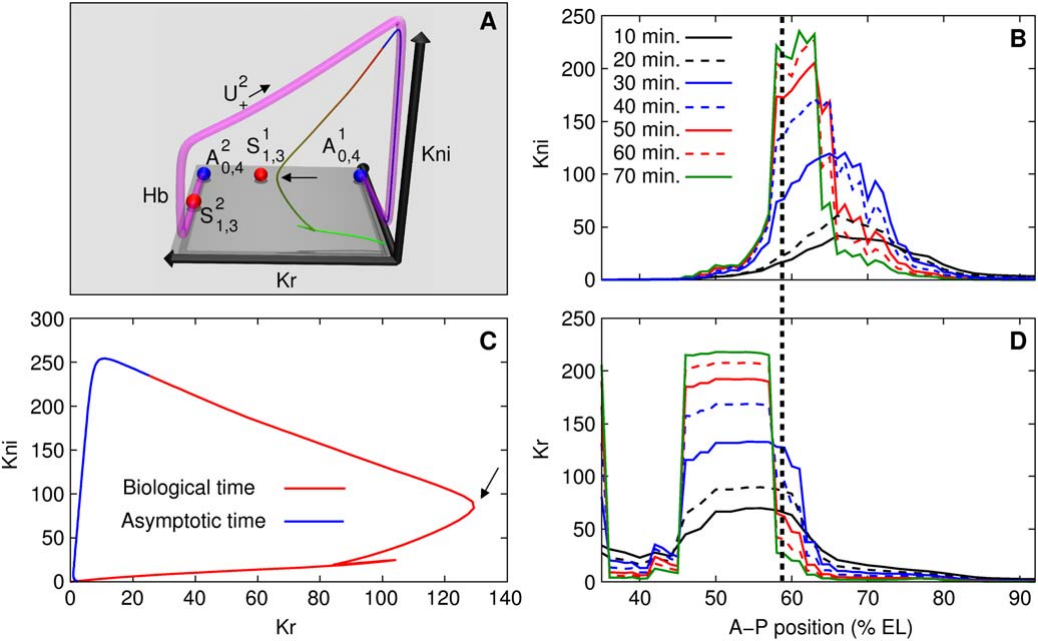
Shifts due to attraction by the manifold 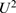. (B,D) Dynamics of protein concentrations in the nucleus at 59% EL, through which the Kr posterior and Kni anterior boundaries pass as they shift to the anterior. The nucleus at 59% EL is indicated with a dashed vertical line. (A) The Hb-Kr-Kni projection of the phase portrait of the nucleus. The invariant manifold 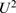 is shown as a magenta tube. The trajectory in the nucleus is plotted in a continuous color gradient from green (*t* = 0 min) to red (*t* = 71.1 min, gastrulation). Times after gastrulation are depicted as blue. The nucleus passes through intermediate states (indicated with an arrow) with high Kr concentrations before reaching a state with high Kni concentration by the onset of gastrulation. This registers as an anterior shift in the posterior *Kr* and anterior *kni* borders. (C) A two dimensional projection of the trajectory in the Kr, Kni plane. The trajectory (red) starts at the origin. It attains a high Kr value at *t* = 45 min (arrow) before approaching high Kni values. The temporary reversal in the trajectory is a mitosis, during which the trajectory moves toward the origin. Time after gastrulation is shown in blue.

Finally, the attraction of trajectories by the manifold 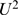 provides a mechanism for dynamical shifts of gap gene domains [21]. We illustrate this mechanism with the nucleus through which the Kr posterior border and the Kni anterior border pass as they shift anteriorly (Fig. 6B,D). Fig. 6A shows the trajectory of the nucleus at 59% EL. The trajectory starts on the Hb axis, and is attracted to 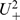, reaching close to the *kni*-on state at gastrulation. However in approaching 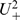, it goes through intermediate states. First Kr increases, and then Kni increases with a concomitant reduction in Kr (see Fig. 6C). We note that the 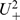 trajectory is a result of asymmetric repression of anterior domain genes by posterior domain proteins [21]. A nucleus in the Kr-on state goes to the Kni-on state because Kni's repression of *Kr* is stronger than that of *kni* by Kr. A similar mechanism applies to nuclei at the Kni posterior and Gt anterior boundaries (data not shown).

## Discussion

**Figure 7:**
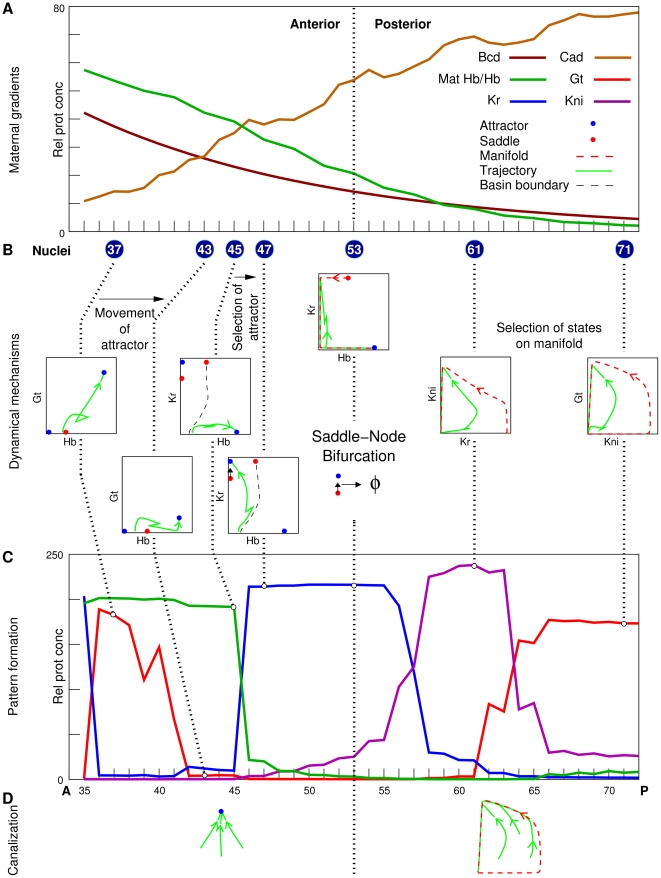
Summary of the dynamical mechanisms of canalization and pattern formation. (A) Maternal gradients in the analyzed region 35%–71% EL. (B) Dynamical mechanisms. 2D phase portraits and trajectories of highlighted nuclei are shown. Dotted lines connect the highlighted nuclei and their phase portraits with the gap gene state shown in panel C. (C) Gap gene expression patterns (T8) in the absence of diffusion and *tll*. (D) Dynamical mechanisms of canalization.

A discrete [6],[7] and buffered response to perturbations is the hallmark of a canalized developmental system. Without recourse to molecular data, Waddington sought to explain these two properties of the response by postulating certain favored stable developmental trajectories which he called chreods. Our results (see Fig. 7 for summary) show that dynamical systems with multiple attracting states possess both of these properties. Small perturbations are damped because of phase space volume contraction driven by attractors. A discrete response to larger perturbations is a consequence of the discontinuous boundaries between the basins of attraction of a multistable system or of bifurcations. Using a model based on gene expression data, we can conclude that the trajectory of the gap gene system is a chreod.

The initial high variation of gap gene expression may arise from early events governed by stochastic laws. Previous observations indicate that the first nuclei in which gap gene transcription is activated are selected probabilistically [68],[76]. Moreover, the gradients of Hb and Cad proteins are formed by translational repression from the Nanos and Bcd gradients respectively [57],[77],[78] under conditions of relatively low molecular number [23], which is likely to lead to intrinsic fluctuations [79]. Our results show that a deterministic description of gap gene dynamics is sufficient to account for the reduction of initial variation regardless of its source. It is evident however that there are at least two other types of variation that the system might be subject to. First, a natural population will have genotypic variation which, in the framework of the model, would be reflected in the variation of its parameters. Second, gap gene expression itself is likely to be a stochastic process rather than a deterministic one. Notwithstanding this fact, there is no evidence in *Drosophila* for the coupling of molecular fluctuations to phenotypic fluctuations as seen in prokaryotes [80], suggesting that molecular fluctuations are buffered in some sense. We emphasize that an attractor is stable against small perturbations of the model itself [29], and hence is a model property that is preserved to an extent if there is genotypic variation in a population or if errors are introduced by stochastic gene expression. However, further study of both of these aspects of canalization is required in order to more fully understand their role.

With regard to pattern formation in the blastoderm, the prevailing theory is that the border positions of downstream genes are determined at fixed values or thresholds of the Bcd gradient [23],[52]. This idea cannot, however, account for either the low variability of downstream gene border positions [14],[17],[18], or the dynamical shifts of domains in the posterior [14],[21]. Fixed threshold specification also cannot explain precise placement of the borders in the posterior since the low molecular number of Bcd in the nuclei implies a high level of molecular noise [23],[81]. In the dynamical picture (Fig. 7), contrary to the threshold view, Bcd ceases to have a role in positional specification posterior to the peak of the *Kr* domain since, posterior to this position, the geometry of the phase space does not change qualitatively with A–P position. Instead, maternal Hb acts as a morphogen, obviating the problems arising from a low molecular number of Bcd. Maternal Hb has long been recognized as a morphogen [74],[75] for the posterior region but the mechanism with which it specifies the posterior region pattern was not clear. As is the case with Bcd, a threshold-based theory for positional specification by Hb [82] is incomplete and requires the postulation of thresholds that can be modified by their targets. The qualitative dynamics provides a viable mechanism for posterior patterning. The attracting manifold 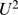 is the geometric manifestation of asymmetric repression between the gap genes in reverse order of gap gene domains, 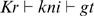. The initial Hb concentration determines which neighborhood of the manifold the trajectory traverses as it is reaches the manifold: *Kr*-on, *kni*-on, or *gt*-on. In other words, posterior patterning works by triggering particular feedback loops in the gap gene network based on maternal Hb concentration. This mechanism also accounts for domain shifts, a property particular to the posterior region, since the trajectories mimic the geometry of the manifold as they approach it.

The dynamical analysis of the gap gene system provides a simple and integrative view of pattern formation in the blastoderm (Fig. 7). The existence of distinct anterior and posterior patterning systems was inferred from the effect of maternal mutations on larval cuticle phenotype and was subsequently characterized in terms of the effects of the Bcd [52],[73],[83],[84] and maternal Hb gradients [56],[57],[77]. But where and how is the control of patterning transferred from Bcd to maternal Hb? Our analysis shows that the hand-off occurs at the A–P position where the *Kr*-on attractor is annihilated through a saddle-node bifurcation, implying a sharp rather than gradual transfer. With knowledge of the two dynamical regimes, the complex spatiotemporal dynamics of the gap gene system can be understood in the simple terms of three mechanisms: movement of attractors through phase space, selection of attractors by initial conditions, and the selection of states on an attracting manifold (Fig. 7).

Finally, we mention the advantage of having the unexpected mechanism of a one dimensional manifold for canalization and patterning. The Bcd concentration is a bifurcation parameter of the dynamical equations. If there were specific attractors corresponding to each gap gene state, with bifurcations creating and annihilating them successively as the Bcd concentration is varied, the molecular noise in Bcd [23] would give rise to “jitter” or rapid switching between attractors. The manifold with its smooth dependence on maternal Hb is qualitatively robust to such fluctuations. In a connectionist model of cognition [85], one dimensional unstable manifolds connecting a sequence of saddle points have been proposed as a means of representing transient brain dynamics. The gap gene phase space is a low dimensional projection of the high dimensional phase space of all the molecular determinants in the blastoderm. It may well be that the attractors found in our analysis are actually saddle points in the high dimensional phase space and are way points, with manifolds connecting them, rather than final end points.

## Methods

The methods used to obtain and characterize the quantitative data are as described in earlier work [14]. All gene expression levels are on a scale of 0–255 chosen to maximize dynamic range without saturation. The numerical implementation of the gene circuit equations is as described [18],[21]. The gap gene circuit was fit to integrated gap gene data [14] using Parallel Lam Simulated Annealing (PLSA) [86],[87]. PLSA minimizes the root mean squared (RMS) difference between model output and data. For each nucleus, data were available at nine time points (Table S1). Search spaces, penalty function, and other annealing parameters were as described [22],[88]. The circuit analyzed in detail had an RMS score of 10.76, corresponding to a proportional error in expression residuals of about 4–5%.

Equilibria were determined by the Newton-Raphson method as described in Protocol S3. One-dimensional unstable manifolds of hyperbolic equilibria were calculated by solving the ODEs using the Bulirsch-Stoer [71] method with starting points in the unstable eigenspace of the equilibria [72]. The basin boundaries on the Hb axis were calculated by finding starting points for trajectories that reach saddle points with one positive eigenvalue (Protocol S3). The time evolution of volume phase space was calculated as described (Protocol S8). The methods used to calculate the equilibria branches and to determine the type of bifurcations are described in Protocol S4.

## Supporting Information

Protocol S1 Hybrid nonautonomous and autonomous equations.
(0.03 MB PDF)

Protocol S2 Equilibria and bifurcations in two dimensions.
(0.02 MB PDF)

Protocol S3 Equilibria, stability, one-dimensional manifolds, and basins of attraction.
(0.04 MB PDF)

Protocol S4 Continuous analysis and bifurcations.
(0.03 MB PDF)

Protocol S5 Saddle equilibria, bifurcations, and basins in the anterior region.
(0.02 MB PDF)

Protocol S6 Saddle equilibria, bifurcations, and basins in the posterior region.
(0.02 MB PDF)

Protocol S7 Reduction of variation in maternal Hb.
(0.02 MB PDF)

Protocol S8 The calculation of volume changes over time.
(0.03 MB PDF)

Figure S1 Integrated data for time-varying inputs. The data are from cleavage cycles 12 (C12), 13 (C13), and 14 (T1–T8). (A) Cad. (B) Tll; T4 and T5 curves are underneath the T6 curve at the peak of the posterior domain. Shaded area shows modeled region.
(0.21 MB TIF)

Figure S2 Equilibria and bifurcations in two dimensions.
(0.06 MB TIF)

Figure S3 Interpolation of time class T6 Cad profile for continuation analysis. The interpolant (black curve) is the cubic polynomial *−0.0075x^3^+0.2264x^2^+2.3611x+9.8004*.
(0.15 MB TIF)

Figure S4 Other equilibria branches determined by the continuous analysis. Saddle equilibria having one or two eigenvalues with positive real part are red or brown respectively. The *y*-axis is the projection of equilibria positions on the Kr axis. The *x*-axis is the bifurcation parameter, the A–P position *x*. (A) The equilibria *S^3^_1,3_* and *S^4^_2,2_*, showing their bifurcation at *36.96%* EL. (B) *S^7^_1,3_* and *S^8^_2,2_* are created at *53.32%* EL, and there are no further bifurcations at more posterior positions.
(0.14 MB TIF)

Figure S5 Bifurcations in the anterior region. Hb-Kr-Gt projection of equilibria diagrams at (A) 35% EL, (B) 37% EL, (C) 43% EL, and (D) 45% EL. The axes originate from *(−10,−10,−10)*, and have length *250* in relative concentration units. The *xy*-plane is shown in gray. To aid perception of depth, shadows from a light source directly above the *xy*-plane are rendered as dark gray traces on the *xy*-plane. Equilibria are represented by spheres of radius *10*. Point attractors are blue and saddle equilibria having one or two eigenvalues with positive real part are red or brown respectively. Red arrows in panel A point to saddles, *S^3^_1,3_* and *S^4^_2,2_*, that disappear through a saddle-node bifurcation between 35% EL and 37% EL. In panels B and C, the *A^1^_0,4_* attractor goes from *hb,gt*-on state to *hb*-on state. Red arrows in panel D point to two saddles, *S^5^_1,3_* and *S^6^_2,2_* created by a saddle node bifurcation between 43% EL and 45% EL. *S^5^_1,3_* and *A^3^_0,4_* disappear through a saddle node bifurcation at 53% EL that separates the anterior and posterior regimes.
(1.80 MB TIF)

Figure S6 All four three-dimensional projections of the phase portrait at 37% EL. (A) Hb-Kr-Gt projection; red arrows are basin boundaries. (B) Hb-Kr-Kni projection. (C) Hb-Gt-Kni projection. (D) Kr-Gt-Kni projection. The axes, *xy*-plane, and equilibria are as in Fig. S5. *10* trajectories are shown with starting points equally distributed on the Hb axis between *0*–*100*. Time is represented as a color gradient along the trajectories, with start of cycle 13 as green, and gastrulation as red; trajectories are blue after gastrulation. The temporary reversals in trajectories are mitoses, during which the trajectories move toward the origin.
(2.22 MB TIF)

Figure S7 Bifurcations in the posterior region. Hb-Kr-Kni projection of phase portraits at (A) 53% EL and (B) 55% EL. The axes, *xy*-plane, and equilibria are as in Fig. S5. See Table S3 for bifurcation parameter values determined by the continuous analysis. Black arrows point to saddles, *S^7^_1,3_* and *S^8^_2,2_*, that are created via a saddle-node bifurcation between 53% EL and 55% EL.
(0.93 MB TIF)

Figure S8 All four three-dimensional projections of the phase portrait at 57% EL. Axes, *xy*-plane, and equilibria are as in Fig. S5. All saddle equilibria are not shown (see Fig. S7). The unstable manifold of saddle *S^2^_1,3_*, *U^2^* is shown as a translucent magenta tube of radius *5*. 10 trajectories are shown in panels A, C, and D, while 25 are shown in panel B. (A) Hb-Kr-Gt projection. Red arrow shows the separation of the indirect route trajectories from direct route ones. (B) Hb-Kr-Kni projection. Red arrow shows the separation of the indirect route trajectories from direct route ones. (C) Hb-Gt-Kni projection. (D) Kr-Gt-Kni projection. *U^2^_+_* traverses the anteroposterior progression of gap gene states in the posterior region—*Kr*-on to *kni*-on to *gt*-on.
(2.69 MB TIF)

Figure S9 Tolerance to variation in maternal Hb. The range of initial conditions (B, error bars) for which modeled gap gene expression patterns have the same expression level variation as gap gene data in T8 (A). Error bars are ranges of concentrations, and percentage variation is the ratio of range to mean. (A) The variation in expression levels at the Kr peak is 30% (yellow bar), at the Kni peak is 35% (red bar), and at the Gt peak is 50% (black bar). (B) The tolerance range for maternal Hb is shown at three A–P positions (*Kr*, *kni*, and *gt* peaks). Maternal Hb profile is shown in red. The tolerance to initial variation is *150%* at *Kr* peak, *85%* at *kni* peak and *100%* at *gt* peak.
(0.46 MB TIF)

Table S1 Time classes
(0.02 MB PDF)

Table S2 Position of gap gene boundaries in the circuits with and without diffusion.
(0.01 MB PDF)

Table S3 Comparison of bifurcation parameter values determined in the discrete analysis with the values determined in the continuous analysis.
(0.02 MB PDF)

Table S4 Summary of all equilibria, the A–P region they exist in, and their function.
(0.02 MB PDF)
