# Supplementary material for: Canalization of Gene Expression and Domain Shifts in the Drosophila Blastoderm by Dynamical Attractors
Source: PLoS Comput Biol. 2009 Mar 13;5(3):e1000303. doi: 10.1371/journal.pcbi.1000303 (PMC2646127; doi:10.1371/journal.pcbi.1000303)
Supplement: Protocol S1 — Hybrid nonautonomous and autonomous equations. (0.03 MB PDF) [file pcbi.1000303.s001.pdf]

S1 Hybrid nonautonomous and autonomous equations

In the Stability Analysis of the Trajectories of the Gap Gene System section the full 232-dimensional gap gene circuit equations were reduced to 58 independent 4-dimensional systems of ODEs (Eq. 3) parametrized by Bcd and Cad concentrations. Since the concentration of Cad changes with time, these equations are nonautonomous and the equilibria in the phase space of a nucleus change with time. The equilibria were calculated at time class T6 since we are interested in the behavior of the system close to gastrulation. The trajectories were calculated using hybrid nonautonomous and autonomous equations so that their asymptotic behavior is governed by the equilibria. Eq. (3) was rewritten such that the system is nonautonomous until time class T6 and autonomous thereafter,

$$\frac{dv^a}{dt} = \begin{cases} R^a g\left(\sum_{b=1}^{N} T^{ab} v^b + m^a v^{\mathrm{Bcd}}(x) + E^{a\leftarrow \mathrm{Cad}} v^{\mathrm{Cad}}(x,t) + h^a\right) \\ \qquad -\lambda^a v^a, \quad \text{if} \quad t < t_6, \\ R^a g\left(\sum_{b=1}^{N} T^{ab} v^b + m^a v^{\mathrm{Bcd}}(x) + E^{a\leftarrow \mathrm{Cad}} v^{\mathrm{Cad}}(x,t_6) + h^a\right) \\ \qquad -\lambda^a v^a, \quad \text{if} \quad t \geq t_6. \end{cases} \tag{S1}$$

Here, t_6 is the midpoint of time class T6 (see Table S1). Note that for $t \geq t_6$, $v^{\mathrm{Cad}}(x,t) \equiv v^{\mathrm{Cad}}(x,t_6)$, that is, the concentration of Cad does not change after T6.

Equilibria were calculated as points $\hat{v}^a$ that satisfy the condition

$$\frac{d\hat{v}^a}{dt} = 0, \quad \text{for} \quad t \geq t_6. \tag{S2}$$

This is equivalent to setting the autonomous part of Eq. (S1) to zero. Let $\hat{v}^{\mathrm{Cad}} = v^{\mathrm{Cad}}(x,t_6)$. Then the equilibria $\hat{v}^a$ are the solutions of a system of nonlinear coupled equations, given by

$$R^a g\left(\sum_{b=1}^{N} T^{ab}\hat{v}^b + m^a v^{\mathrm{Bcd}}(x) + E^{a\leftarrow \mathrm{Cad}}\hat{v}^{\mathrm{Cad}}(x) + h^a\right) - \lambda^a \hat{v}^a = 0. \tag{S3}$$
