# Supplementary material for: Canalization of Gene Expression and Domain Shifts in the Drosophila Blastoderm by Dynamical Attractors
Source: PLoS Comput Biol. 2009 Mar 13;5(3):e1000303. doi: 10.1371/journal.pcbi.1000303 (PMC2646127; doi:10.1371/journal.pcbi.1000303)
Supplement: Protocol S2 — Equilibria and bifurcations in two dimensions. (0.02 MB PDF) [file pcbi.1000303.s002.pdf]

S2 Equilibria and bifurcations in two dimensions

Close to a typical equilibrium point, there exist principal directions or planes in which the solution either approaches or moves away from the equilibrium at an exponential rate. These directions are called eigenvectors of the equilibrium, and the exponential rates are called eigenvalues. It is possible that the solution either spirals toward or away from the equilibrium point in a two-dimensional plane instead of a single direction. In this case the eigenvalue is a complex number and the distance between the solution and the equilibrium changes at an exponential rate given by the real part of the eigenvalue. For simplicity the following discussion is restricted to real-valued eigenvalues though equilibria with complex eigenvalues have similar properties based on the value of the real part of the eigenvalue [1]. An eigenvalue with a negative value implies that the solution approaches the equilibrium along the eigenvector, while an eigenvalue with a positive value implies that the solution moves away from the equilibrium along the eigenvector. Equilibria can have different stability properties depending on their eigenvalues [1, 2].

In a system with two state variables three cases are possible, as illustrated in the top three panels of Fig. S2. An *attractor* or *node* equilibrium [1] has two negative eigenvalues, and hence attracts all solutions in a surrounding region. This region is called the basin of attraction of the equilibrium. While the eigenvalues provide information about the solutions close to the attractor, the basin of attraction determines the behavior of solutions farther away from the attractor. A *repeller* [1] has two positive eigenvalues and all solutions in its neighborhood move away from it.

A *saddle* equilibrium has one negative and one positive eigenvalue. The eigenvectors are shown as arrows. The eigenvector associated with the negative eigenvalue extends to a curve called the stable manifold (S) [2], which is the only solution that approaches the equilibrium. All other solutions eventually move away from the saddle point. The eigenvector associated with the positive eigenvalue extends to a curve called the unstable manifold (U) [2], which is the only solution that would approach the equilibrium if the arrow of time were reversed. In a higher-dimensional system such as the gap gene system saddles with more than one negative eigenvalue are possible, and their unstable and stable manifolds are surfaces of dimension equal to the number of positive and negative eigenvalues respectively. As is the case with the basin of an attractor, the manifolds of a saddle point determine the behavior of solutions farther away from it.

If the system of equations depends on a parameter such as the A–P position in the gap gene system, the equilibrium solutions change with the parameter. Of particular interest are changes in the number of equilibria or their stability, called bifurcations. Bifurcations cause a qualitative change in the dynamics of the system. They occur when the system has an equilibrium point with one or more zero eigenvalues (a *degenerate* equilibrium [1]). The bottom three panels of Fig. S2 (from left to right) illustrate a saddle-node bifurcation in a two-dimensional phase space. At less

than a critical value of the parameter, the system has a saddle and a node. At the critical value, there is only one equilibrium, which is degenerate. At values of the parameter greater than the critical value, there are no equilibria in the system.

References

[1] Hirsch M, Smale S, Devaney R (2004) Differential Equations, Dynamical Systems, and an Introduction to Chaos. Boston: Academic Press.

[2] Perko L (1996) Differential Equations and Dynamical Systems. New York: Springer-Verlag.
