# Supplementary material for: Canalization of Gene Expression and Domain Shifts in the Drosophila Blastoderm by Dynamical Attractors
Source: PLoS Comput Biol. 2009 Mar 13;5(3):e1000303. doi: 10.1371/journal.pcbi.1000303 (PMC2646127; doi:10.1371/journal.pcbi.1000303)
Supplement: Protocol S3 — Equilibria, stability, one-dimensional manifolds, and basins of attraction. (0.04 MB PDF) [file pcbi.1000303.s003.pdf]

S3 Equilibria, stability, one-dimensional manifolds, and basins of attraction

This section describes the calculation of equilibria, their stability, associated one-dimensional manifolds and basins of attraction.

For the pair of Bcd and Cad concentrations ($v^{Bcd}(x)$ and $\hat{v}^{\mathrm{Cad}}(x)$) that position the nucleus at A–P position x, the equilibria $\hat{v} = (\hat{v}^{\mathrm{Hb}}, \hat{v}^{\mathrm{Kr}}, \hat{v}^{\mathrm{Gt}}, \hat{v}^{\mathrm{Kni}})$ were calculated by solving Eq. (S3) (Protocol S1), using the Newton-Raphson method [1, 2]. The ability of the Newton-Raphson algorithm to find zeroes depends on the starting point. To ensure that all equilibria were obtained, the Newton-Raphson iteration was started from n^4 points on a uniform grid in the four-dimensional box $(0, 250) \times (0, 250) \times (0, 250) \times (0, 250)$. Newton-Raphson was run with a tolerance of 10^{-6}, and only a negligible number of starting points failed to converge. n was increased from 3 to 51. For all nuclei, no new equilibria were found after $n = 10$.

The equilibria were classified according to the eigenvalues of the linearized system of equations $\frac{dy}{dt} = J(\hat{v}^a)y$, where $y = v^a - \hat{v}^a$ is a 4-dimensional vector, and $J(\hat{v}^a)$ is the Jacobian of the autonomous part of Eq. (S1) (Protocol S1) at the equilibrium point $\hat{v}^a$. The eigenvalues are complex numbers in general, and the real part determines the stability of the equilibrium. An equilibrium which has no eigenvalue with zero real part is called a non-degenerate hyperbolic equilibrium. A hyperbolic equilibrium which has all eigenvalues with negative real parts is called an attractor or a node. A hyperbolic equilibrium which has at least one eigenvalue with a positive real part and at least one with a negative real part is called a saddle equilibrium. Degenerate equilibria are defined by J being singular, that is, they have at least one zero eigenvalue. A bifurcation is said to occur when the number of equilibria changes.

A saddle equilibrium has $p < 4$ eigenvalues with positive real parts and $q = 4 - p$ eigenvalues with negative real parts. The jth such point is denoted by $S^j_{p,q}$. p is called the index of the saddle equilibrium. Associated with such a point are two invariant sets, called the global stable manifold and the global unstable manifold [3]. The global stable manifold is the set of all points such that trajectories starting from them have the saddle equilibrium as their limit as $t \to \infty$, and is of dimension $4 - p$. The global unstable manifold is the set of all starting points whose trajectories have the saddle equilibrium as their limit as $t \to -\infty$, and is of dimension p. The stable and unstable manifolds of saddles of index 1 ($S^j_{1,3}$) are of particular interest. The three-dimensional stable manifold of such points forms the boundaries for basins of attraction of point attractors [4]. A stable manifold of dimension 3 is very computationally expensive to calculate [5, 6]. By comparison, calculating the one-dimensional unstable manifold of $S^j_{1,3}$ is straightforward [4]. Two starting points were chosen, one displaced by 10^{-1} from $S^j_{1,3}$ in the direction of the eigenvector of the positive

eigenvalue; the other displaced by the same amount in the opposite direction. The union of trajectories resulting from these starting points, U_+^j, and U_-^j is an approximation to the one-dimensional unstable manifold of $S_{1,3}^j$.

Note that even though the unstable manifolds of $S_{1,3}^j$ were calculated for the autonomous part of Eq. (S1) (Protocol S1), their definition is more general, and holds for nonautonomous systems of equations as well. The same holds true for stable manifolds.

An equilibrium point that has all eigenvalues with negative real parts is called a node or a point attractor [3, 7]. The jth such point is denoted by $A_{0,4}^j$. There exists a set of points such that any trajectory starting within it approaches the node as $t \to \infty$ [3]. This set is called the basin of attraction of the node, and the node is an attractor of this set.

Since the Hb axis forms the biological set of initial conditions (no Kr, Gt, and Kni protein is detected before cycle 13, see the Gap Gene Circuits section), we only characterized the intersection of the Hb axis with the basin of attraction of the nodes. In this paper the term "basin of attraction" is used with the special meaning that it is the intersection of the four-dimensional basin of attraction with the Hb axis. The trajectories were calculated using the hybrid nonautonomous-autonomous system (Eq. S1) (Protocol S1). They were started from uniformly spaced starting points on the Hb axis and integrated until $|v_i^a(t) - A_{0,4}^j| < 10^{-6}$. The set of starting points that came close to $A_{0,4}^j$ formed the preliminary characterization of its basin.

Next the open interval (B_1^j, B_2^j) on the Hb axis was explicitly calculated, within which all starting points reached the point attractor $A_{0,4}^j$. B_1^j and B_2^j are starting points (on the Hb axis) of singular trajectories that reach close to a saddle equilibrium point of index 1. The set of all singular trajectories that reach a saddle equilibrium of index 1 defines the boundary between basins of attraction [4] of the attractors of the system. Thus B_1^j and B_2^j are the intersection of the basin boundaries with the Hb axis.

References

[1] Conte SD, de Boor C (1980) Elementary Numerical Analysis. New York, New York: McGraw-Hill Book Company.

[2] Press WH, Teukolsky SA, Vetterling WT, Flannery BP (1992) Numerical Recipes in C. Cambridge, U.K.: Cambridge University Press, second edition.

[3] Perko L (1996) Differential Equations and Dynamical Systems. New York: Springer-Verlag.

[4] Guckenheimer J, Vladimirsky A (2004) A fast method for approximating invariant manifolds. SIAM Journal of Applied Dynamical Systems 3:232–260.

[5] Krauskopf B, Osinga H (2005) A survey of methods for computing (un)stable manifolds of vector fields. International Journal of Bifurcation and Chaos 15:763–791.

[6] Dellnitz M, Froyland G, Junge O (2001) The algorithms behind GAIO - set oriented numerical methods for dynamical systems. In: Fiedler B, editor, Ergodic Theory, Analysis, and Efficient Simulation of Dynamical Systems. pp. 145–174.

[7] Hirsch M, Smale S, Devaney R (2004) Differential Equations, Dynamical Systems, and an Introduction to Chaos. Boston: Academic Press.
