# Supplementary material for: Canalization of Gene Expression and Domain Shifts in the Drosophila Blastoderm by Dynamical Attractors
Source: PLoS Comput Biol. 2009 Mar 13;5(3):e1000303. doi: 10.1371/journal.pcbi.1000303 (PMC2646127; doi:10.1371/journal.pcbi.1000303)
Supplement: Protocol S4 — Continuous analysis and bifurcations. (0.03 MB PDF) [file pcbi.1000303.s004.pdf]

S4 Continuous analysis and bifurcations

This section describes the calculation of equilibria branches by a continuous analysis and the characterization of the bifurcations that occur in the gap gene system.

x was varied continuously in Eq. (S3) (Protocol S1) using the polynomial approximation for Cad data (Fig. S3) and the exponential approximation for Bcd ($v^{\mathrm{Bcd}}(x) = A\exp(-\lambda x)$). The bifurcations were detected when the determinant of the Jacobian became zero. The bifurcations determined in this manner cross-validated the discrete analysis of Protocol S3—the two analyses found the same equilibria and bifurcations (Fig. 3 and S4) with exceptions which do not affect the cross-validation. Specifically, $S^9_{1,3}$ exists for such a small interval of x that it was overlooked in the discrete analysis, and hence in the discrete analysis it appeared that $S^6_{2,2}$ and $S^{10}_{2,2}$ were identical. Since the continuation analysis and the discrete analysis used different Cad profiles (Fig. S3), the A–P positions at which these bifurcations occur are slightly different. The comparison is shown in Table S3.

We determined the type of the bifurcation by a center manifold analysis. The bifurcations occur at a degenerate equilibrium, where the determinant of the Jacobian is zero. The center manifold is the invariant manifold with respect to the dynamics passing through the degenerate equilibrium and whose tangent space is the eigenspace corresponding to the zero eigenvalue [1, 2]. In order to contain the bifurcating equilibria branches, the center manifold is extended in the directions of variation of the parameters. The above definition accounts for this extension if the directions of variation of the parameters are included in the zero eigenspace. Thus, the dimension of the center manifold is equal to the degeneracy of the zero eigenvalue of the Jacobian plus the number of parameters [2].

All degenerate equilibria that were detected in the analyzed region (Fig. 3A and S4) have only one zero eigenvalue. The position x is the single bifurcation parameter. Thus, the center manifold has dimension two. In this case the orbit structure on the center manifold is very simple. Only three situations are possible [2]: a) a saddle-node bifurcation, b) a transcritical bifurcation, or c) a pitchfork bifurcation. In our case it is easy to identify the saddle-node bifurcation without explicit calculation of the normal form, because this is the only situation among the three possibilities when a unique curve of equilibria passes through the degenerate equilibrium. For the transcritical and pitchfork bifurcations there are two such curves.

This curve folds at the bifurcation, such that a repeller and a node coexist on one side of the bifurcation value of the parameter, with no fixed point on the other side. We have also checked that the orbit structure corresponds to a saddle-node bifurcation by showing that there is a trajectory that starts in the unstable eigenspace of one equilibrium point and reaches the other equilibrium point. This trajectory connects the repeller to the node on the center manifold. For example, for the

bifurcation in which $S^5_{1,3}$ and $A^3_{0,4}$ disappear (Fig. 3A), the center manifold contains the trajectory from the unstable eigenspace of $S^5_{1,3}$ that reaches $A^3_{0,4}$. This trajectory becomes shorter and shorter and reduces to the degenerate fixed point at the saddle-node bifurcation. By applying this analysis to all the bifurcations, we found that they were all of saddle-node type.

References

[1] Perko L (1996) Differential Equations and Dynamical Systems. New York: Springer-Verlag.

[2] Wiggins S (1990) Introduction to Applied Nonlinear Dynamical Systems and Chaos. New York: Springer-Verlag.
