# Supplementary material for: Canalization of Gene Expression and Domain Shifts in the Drosophila Blastoderm by Dynamical Attractors
Source: PLoS Comput Biol. 2009 Mar 13;5(3):e1000303. doi: 10.1371/journal.pcbi.1000303 (PMC2646127; doi:10.1371/journal.pcbi.1000303)
Supplement: Protocol S5 — Saddle equilibria, bifurcations, and basins in the anterior region. (0.02 MB PDF) [file pcbi.1000303.s005.pdf]

S5 Saddle equilibria, bifurcations, and basins in the anterior region

At 35% EL, there are four saddle equilibria: $S^1_{1,3}$, $S^2_{1,3}$, $S^3_{1,3}$, and $S^4_{2,2}$ (see Fig. S5A). At 36.96% EL, $S^3_{1,3}$ and $S^4_{2,2}$ annihilate each other through a saddle-node bifurcation (see Fig. S4A and compare panels A and B of Fig. S5), leaving only $S^1_{1,3}$ and $S^2_{1,3}$. Two new saddles, $S^5_{1,3}$ and $S^6_{2,2}$, are created by a saddle-node bifurcation (see Fig. 3A and compare panels C and D of Fig. S5) at A–P position 46.14%. $S^9_{1,3}$ and $S^{10}_{2,2}$ are created at 51.72% EL, of which $S^9_{1,3}$ annihilates $S^6_{2,2}$ at 52.05% EL (see Fig. 3A). $S^1_{1,3}$ is contained in the Hb-Kr plane, at the *hb*-on edge (Fig. S5). *Kr* expression at this equilibrium point increases with A–P position, that is, the saddle moves toward the *hb,Kr*-on attractor $A^2_{0,4}$. The $S^2_{1,3}$ saddle equilibrium is also contained in the Hb-Kr plane, at the *Kr*-on edge. With increasingly posterior A–P position, *hb* expression increases; $S^2_{1,3}$ also moves toward $A^2_{0,4}$.

The stable manifolds of the saddle equilibria of index 1 ($S^1_{1,3}$, $S^2_{1,3}$) form the boundaries of the basins of attraction of the point attractors ($A^1_{0,4}$, $A^2_{0,4}$, $A^3_{0,4}$). By calculating singular trajectories from the Hb axis that reach such saddle equilibria, it was found that the basins of $A^1_{0,4}$ and $A^2_{0,4}$ are separated by the stable manifold of $S^1_{1,3}$. Similarly, the basins of $A^2_{0,4}$ and $A^3_{0,4}$ are separated by the stable manifold of $S^2_{1,3}$. The basins of the three attractors in the anterior region were determined as described in Protocol S3 (Table 1).

Except for the anteriormost nucleus (35% EL), the selection of basins of attraction by the concentration of maternal Hb (Table 1) correctly accounts for the observed gap gene expression patterns in all the nuclei of the anterior region. For the anomalous nucleus, the value of maternal Hb puts it in the $A^2_{0,4}$ (*hb,Kr*-on) basin instead of the $A^1_{0,4}$ basin, suggesting that the extent of basins is not correct in this nucleus. This is a minor problem, since the nucleus behaves correctly in the circuit with diffusion (see Fig. 2F).
