# Supplementary material for: Canalization of Gene Expression and Domain Shifts in the Drosophila Blastoderm by Dynamical Attractors
Source: PLoS Comput Biol. 2009 Mar 13;5(3):e1000303. doi: 10.1371/journal.pcbi.1000303 (PMC2646127; doi:10.1371/journal.pcbi.1000303)
Supplement: Protocol S6 — Saddle equilibria, bifurcations, and basins in the posterior region. (0.02 MB PDF) [file pcbi.1000303.s006.pdf]

S6 Saddle equilibria, bifurcations, and basins in the posterior region

The saddles $S^1_{1,3}$, $S^2_{1,3}$, and $S^{10}_{2,2}$ carry over from the anterior region. Two new saddles $S^7_{1,3}$ and $S^8_{2,2}$ are created through a saddle-node bifurcation at 53.32% EL (see Fig. S4B and S7). Following this bifurcation, the qualitative structure of the phase space remains invariant for the rest of the posterior region.

As is the case in the anterior region, the basin boundaries of $A^2_{0,4}$ are formed by the stable manifolds of $S^1_{1,3}$ and $S^2_{1,3}$. Since $A^1_{0,4}$ is the only other attractor, the basin of $A^2_{0,4}$ divides the basin of $A^1_{0,4}$ into two parts (Fig. S8B).
