# Supplementary material for: Canalization of Gene Expression and Domain Shifts in the Drosophila Blastoderm by Dynamical Attractors
Source: PLoS Comput Biol. 2009 Mar 13;5(3):e1000303. doi: 10.1371/journal.pcbi.1000303 (PMC2646127; doi:10.1371/journal.pcbi.1000303)
Supplement: Protocol S7 — Reduction of variation in maternal Hb. (0.02 MB PDF) [file pcbi.1000303.s007.pdf]

# S7 Reduction of variation in maternal Hb

In the anterior region, reduction in initial variation happens due to the convergence of trajectories to a point attractor. The size of an attractor's basin is a natural tolerance range for variation in initial conditions. In the posterior region, since the trajectories are attracted by the one-dimensional manifold $U_+^2$, there is a residual dependence on initial Hb concentration (Fig. S8B). This sensitivity to maternal Hb concentration is required in order to pattern the posterior (Fig. 5). Despite this sensitivity, there is a further reduction of variation in initial conditions. The indirect trajectory interval of $A_{0,4}^1$'s basin (left of the arrow in Fig. S8B) can be divided into subintervals, such that trajectories from within a subinterval reach the same state at gastrulation.

Unlike the anterior region, where basin boundaries provided a natural tolerance range, the posterior phase portraits do not have any feature that can be exploited to calculate these subintervals or tolerances. They were calculated empirically from gap gene variation data in FlyEx. The variation in expression levels of Hb, Kr, Gt, and Kni in time class T8 was determined at three positions on the A–P axis (corresponding to the peaks of Kr, Kni, and Gt). Next, the model was solved with different values of initial Hb concentration as starting points. The tolerance was calculated as the range of Hb initial concentrations that produced model output with the same variation in expression levels as data. The results are shown in Fig. S9. It was found that there is significant reduction of maternal Hb variation in the posterior nuclei. For instance, at Kr peak, where *Kr* expression level varies by $30\%$ in time class T8, the tolerance for initial variation is $150\%$.
