# Supplementary material for: Canalization of Gene Expression and Domain Shifts in the Drosophila Blastoderm by Dynamical Attractors
Source: PLoS Comput Biol. 2009 Mar 13;5(3):e1000303. doi: 10.1371/journal.pcbi.1000303 (PMC2646127; doi:10.1371/journal.pcbi.1000303)
Supplement: Protocol S8 — The calculation of volume changes over time. (0.03 MB PDF) [file pcbi.1000303.s008.pdf]

S8 The calculation of volume changes over time

This section describes a method for calculating the time evolution of an initial volume in the phase space. We used this method to determine the extent of variance reduction in single-nucleus gene circuits (Fig. 4C,D).

Given the diffusionless gap gene dynamical system in the nucleus at A–P position x (Eq. 2)

$$\frac{dv^a}{dt} = R^a g\left(\sum_{b=1}^{N} T^{ab}v^b + m^a v^{\mathrm{Bcd}}(x) + \sum_{\beta=1}^{N_e} E^{a\beta}v^{\beta}(x,t) + h^a\right) - \lambda^a v^a,$$

we want to calculate the volume V_t at time t of a box of initial conditions $\mathcal{B}_0$. Let us represent the right hand side as $f(v_t, t)$, where $v_t = (v_t^1, v_t^2, v_t^3, v_t^4)$ is the four-dimensional state vector at time t.

The ODE is discretized into a map,

$$v_{t+dt} = \gamma(v_t, t), \tag{S1}$$

where $\gamma(v_t, t) = v_t + f(v_t, t)dt$. Regard the map at time $t + (n-1)dt$,

$$v_{t+ndt} = \gamma(v_{t+(n-1)dt}, t + (n-1)dt),$$

as a curvilinear coordinate transformation of the phase space, then the infinitesimal volume at time $t + ndt$,

$$dV_{t+ndt} = dv^1_{t+ndt}dv^2_{t+ndt}dv^3_{t+ndt}dv^4_{t+ndt}$$

can be written as,

$$dV_{t+ndt} = J(v_{t+(n-1)dt}, t + (n-1)dt)dV_{t+(n-1)dt}. \tag{S2}$$

Here, $dV_{t+(n-1)dt}$ is the infinitesimal volume at $t + (n-1)dt$, and $J(v_t, t)$ is the Jacobian of the map at time t. Applying Eq. (S2) repeatedly, the infinitesimal volume at t, dV_t, can be written in terms of the initial infinitesimal volume, dV_0 as

$$dV_t = J(v_{t-(n-1)dt}, t-(n-1)dt)J(v_{t-(n-2)dt}, t-(n-2)dt)\dots J(v_0, 0)dV_0.$$

If the initial box $\mathcal{B}_0$ evolves to $\mathcal{B}_t$ at time t, $\mathcal{B}_t$'s volume is

$$\begin{aligned} V_t &= \iiiint\limits_{\mathcal{B}_t} dV_t \\ &= \iiiint\limits_{\mathcal{B}_0} [J(v_{t-(n-1)dt}, t-(n-1)dt)\dots J(v_0, 0)]dv_0^1 dv_0^2 dv_0^3 dv_0^4. \end{aligned}$$

The integral on the right hand side was evaluated using the multidimensional trapezoidal rule [1], successively refining the grid on the initial box $\mathcal{B}_0$ until the integral converged. The time-step for the discretized map $\gamma(v_t, t)$ was chosen small enough such that the Euler-method solution of Eq. (2) converged.

References

[1] Press WH, Teukolsky SA, Vetterling WT, Flannery BP (1992) Numerical Recipes in C. Cambridge, U.K.: Cambridge University Press, second edition.
