# Supplementary figures and images for: Canalization of Gene Expression and Domain Shifts in the Drosophila Blastoderm by Dynamical Attractors
Source: PLoS Comput Biol. 2009 Mar 13;5(3):e1000303. doi: 10.1371/journal.pcbi.1000303 (PMC2646127; doi:10.1371/journal.pcbi.1000303)

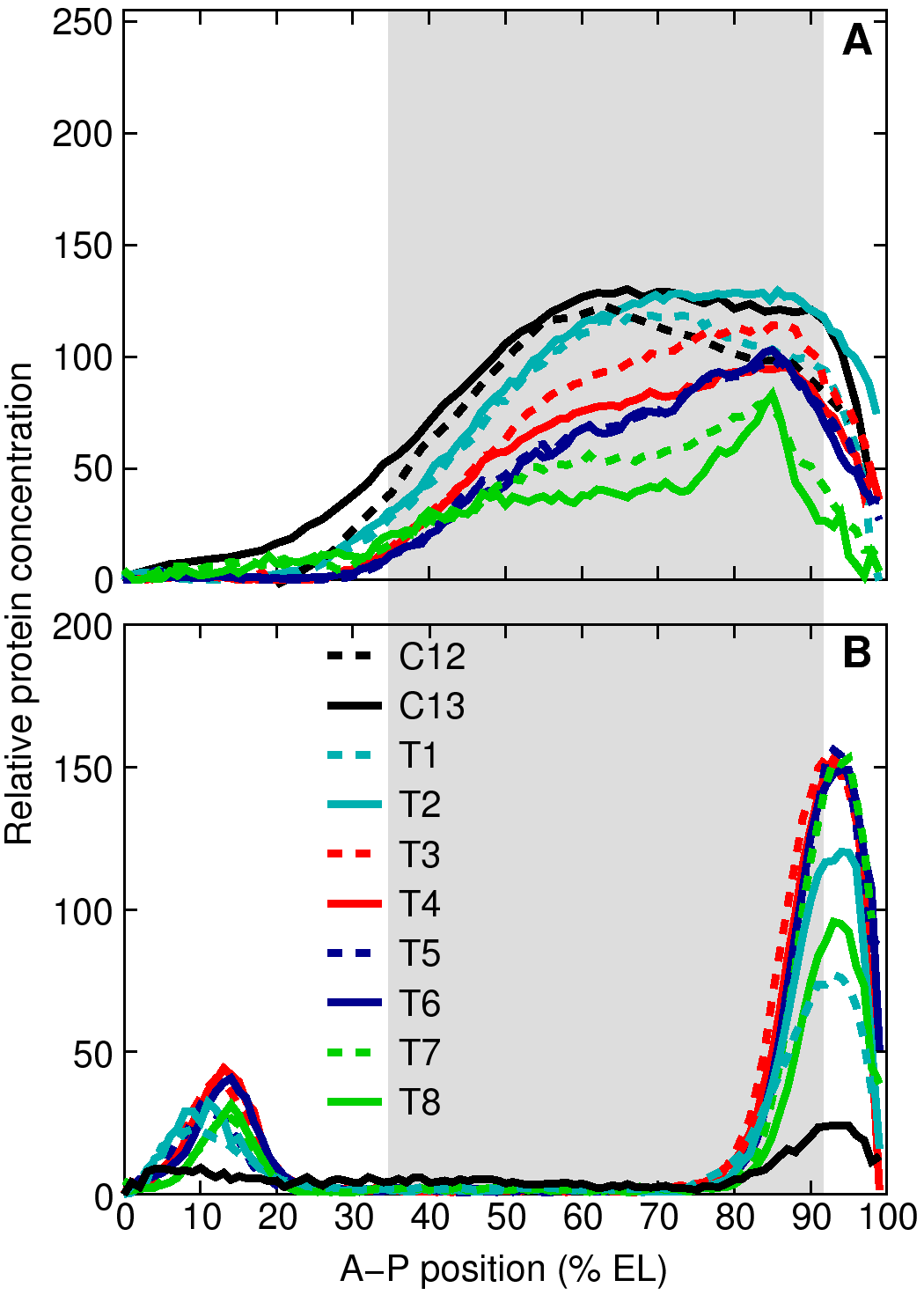

Supplement: Figure S1 — Integrated data for time-varying inputs. The data are from cleavage cycles 12 (C12), 13 (C13), and 14 (T1–T8). (A) Cad. (B) Tll; T4 and T5 curves are underneath the T6 curve at the peak of the posterior domain. Shaded area shows modeled region. (0.21 MB TIF) [file pcbi.1000303.s009.tif]

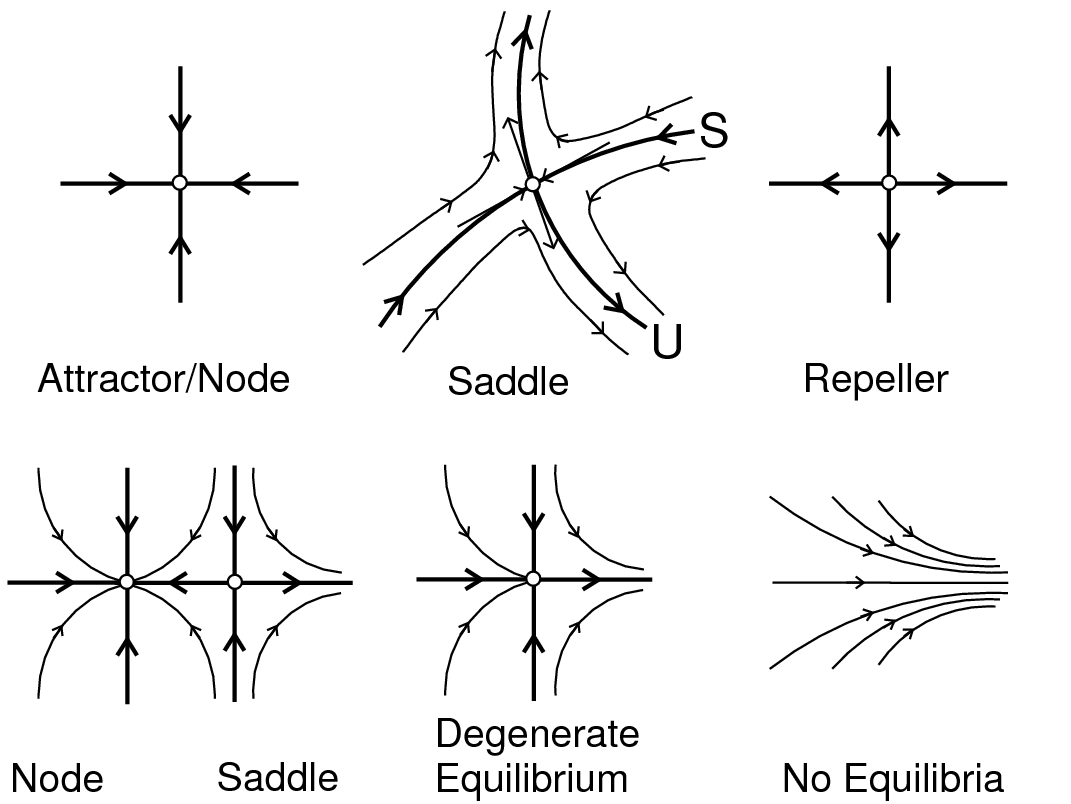

Supplement: Figure S2 — Equilibria and bifurcations in two dimensions. (0.06 MB TIF) [file pcbi.1000303.s010.tif]

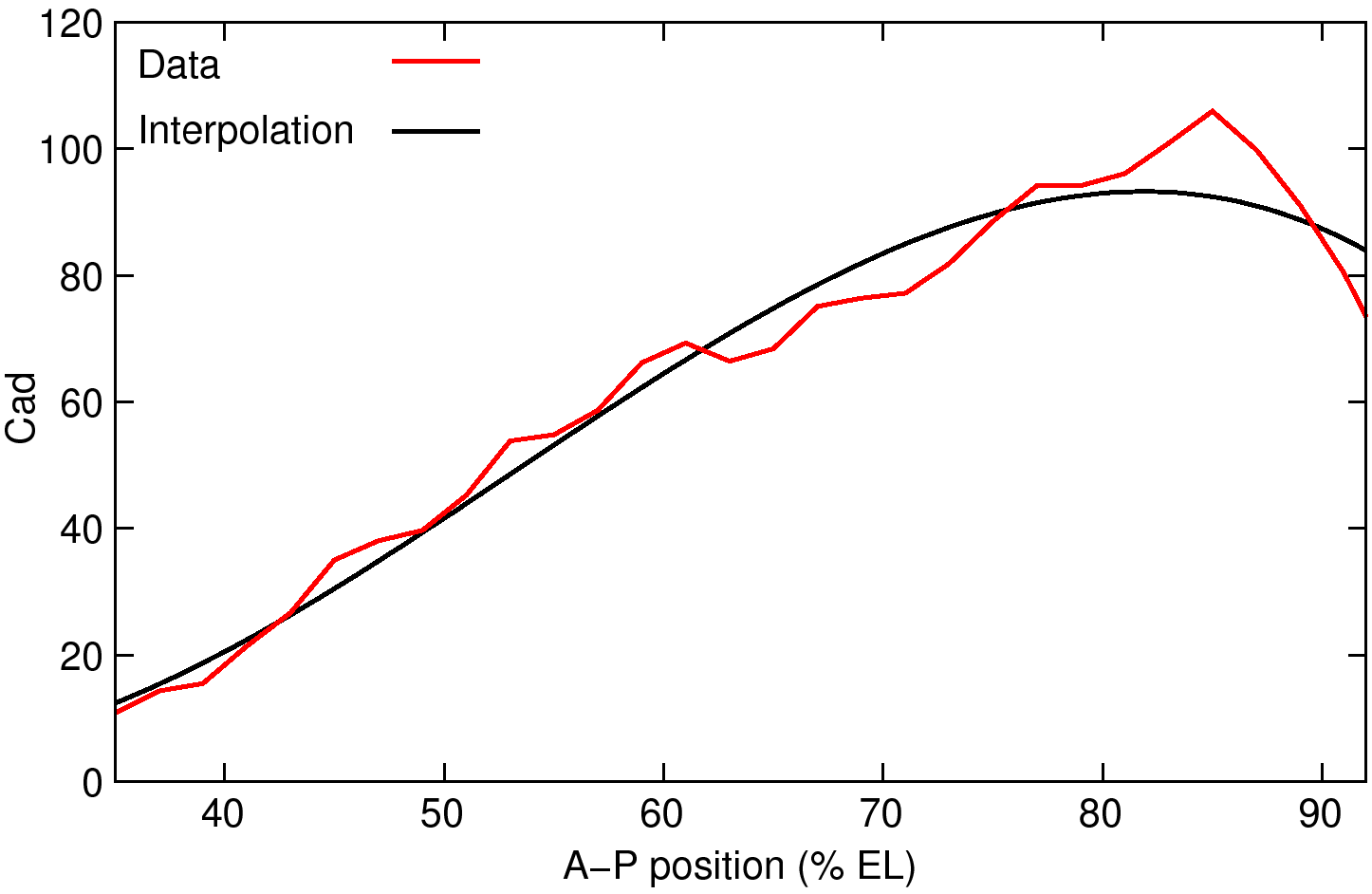

Supplement: Figure S3 — Interpolation of time class T6 Cad profile for continuation analysis. The interpolant (black curve) is the cubic polynomial −0.0075x3+0.2264x2+2.3611x+9.8004. (0.15 MB TIF) [file pcbi.1000303.s011.tif]

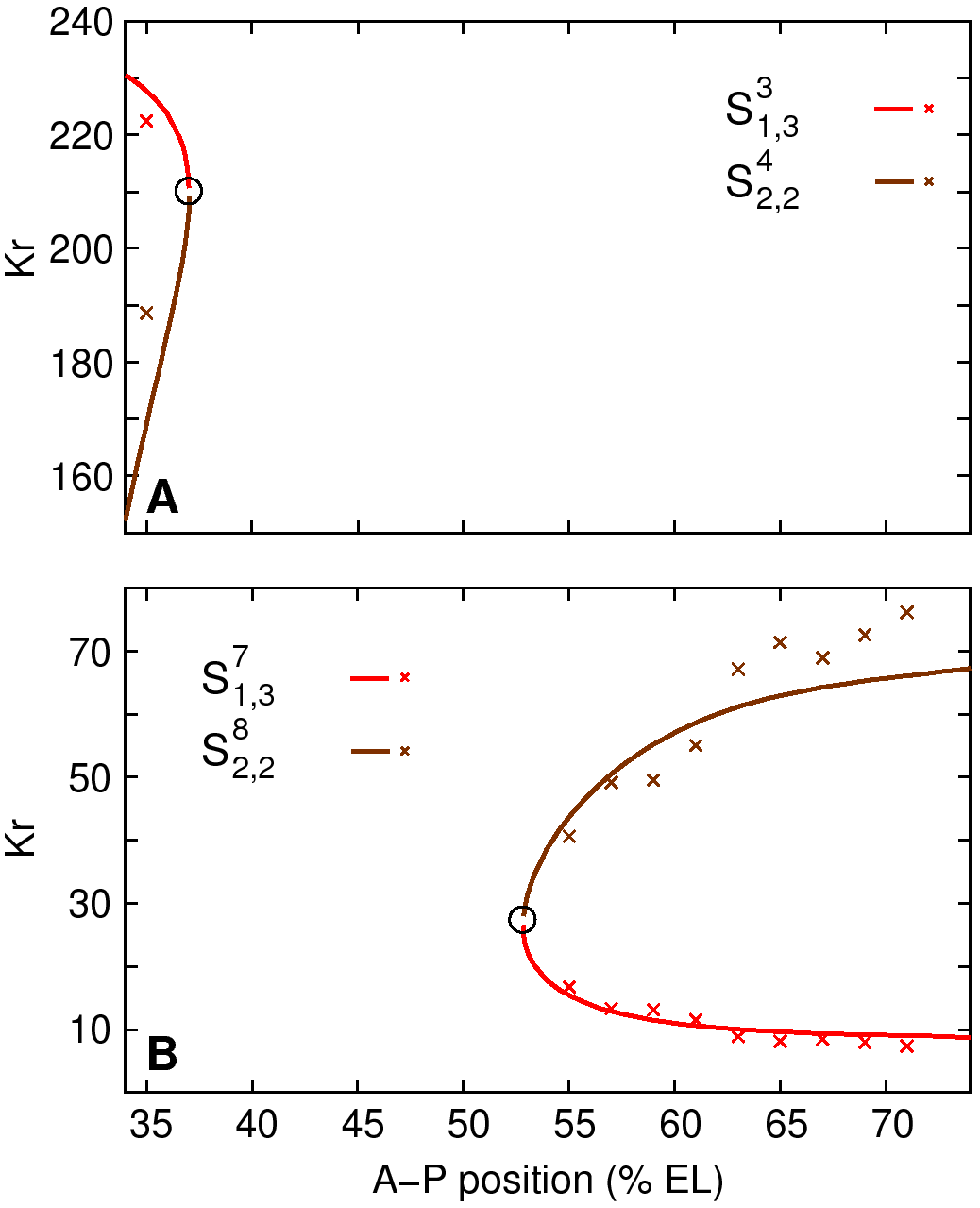

Supplement: Figure S4 — Other equilibria branches determined by the continuous analysis. Saddle equilibria having one or two eigenvalues with positive real part are red or brown respectively. The y-axis is the projection of equilibria positions on the Kr axis. The x-axis is the bifurcation parameter, the A–P position x. (A) The equilibria S31,3 and S42,2, showing their bifurcation at 36.96% EL. (B) S71,3 and S82,2 are created at 53.32% EL, and there are no further bifurcations at more posterior positions. (0.14 MB TIF) [file pcbi.1000303.s012.tif]

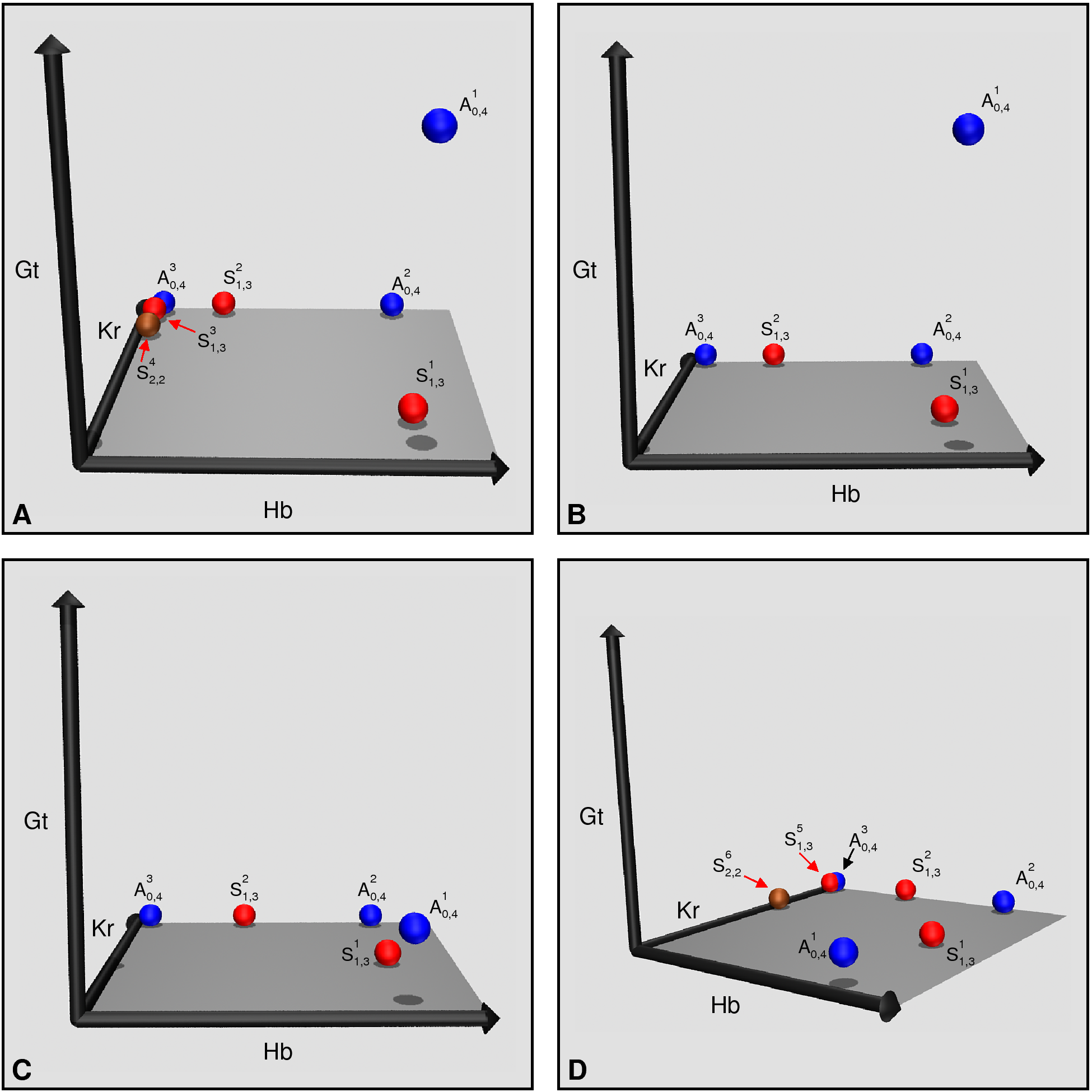

Supplement: Figure S5 — Bifurcations in the anterior region. Hb-Kr-Gt projection of equilibria diagrams at (A) 35% EL, (B) 37% EL, (C) 43% EL, and (D) 45% EL. The axes originate from (−10,−10,−10), and have length 250 in relative concentration units. The xy-plane is shown in gray. To aid perception of depth, shadows from a light source directly above the xy-plane are rendered as dark gray traces on the xy-plane. Equilibria are represented by spheres of radius 10. Point attractors are blue and saddle equilibria having one or two eigenvalues with positive real part are red or brown respectively. Red arrows in panel A point to saddles, S31,3 and S42,2, that disappear through a saddle-node bifurcation between 35% EL and 37% EL. In panels B and C, the A10,4 attractor goes from hb,gt-on state to hb-on state. Red arrows in panel D point to two saddles, S51,3 and S62,2 created by a saddle node bifurcation between 43% EL and 45% EL. S51,3 and A30,4 disappear through a saddle node bifurcation at 53% EL that separates the anterior and posterior regimes. (1.80 MB TIF) [file pcbi.1000303.s013.tif]

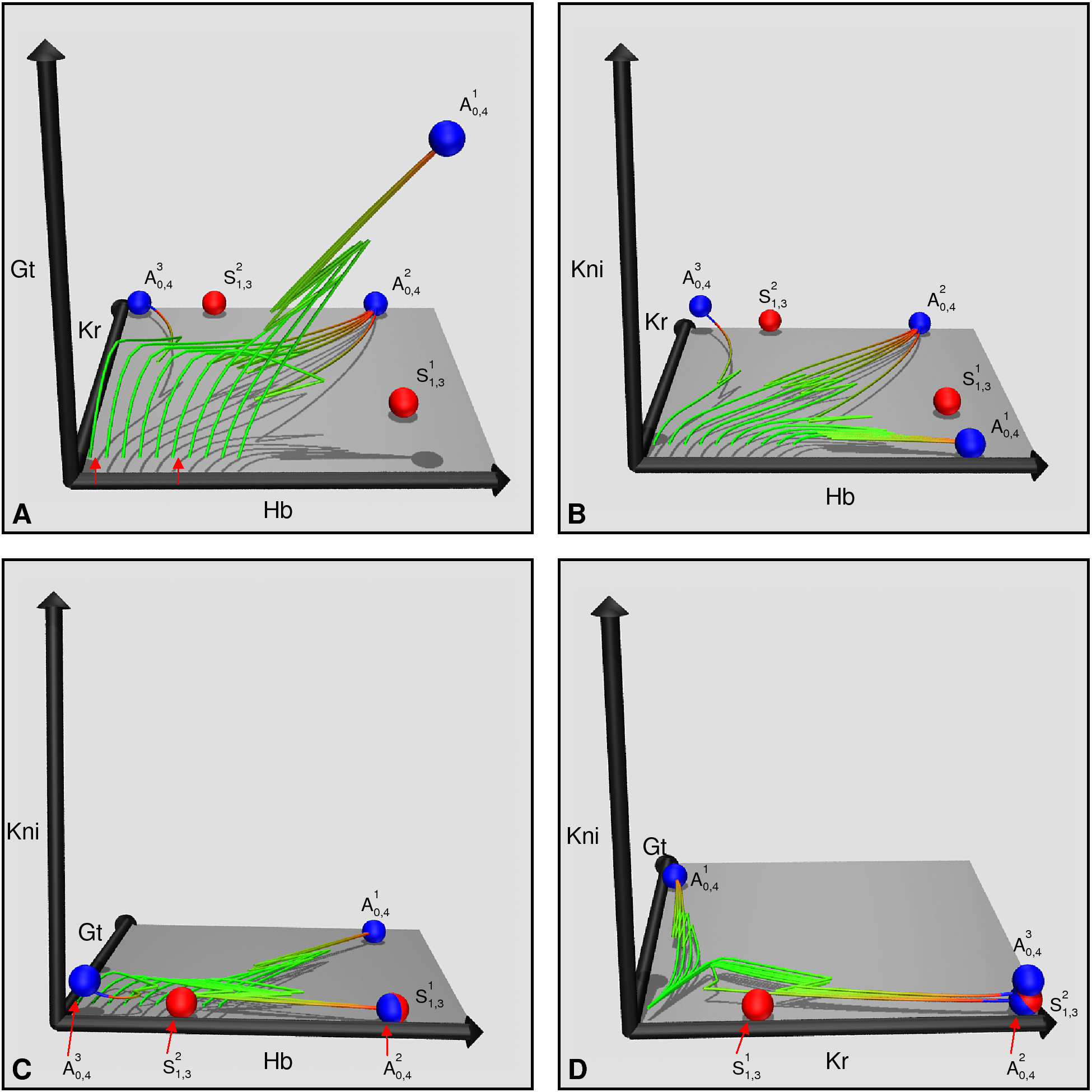

Supplement: Figure S6 — All four three-dimensional projections of the phase portrait at 37% EL. (A) Hb-Kr-Gt projection; red arrows are basin boundaries. (B) Hb-Kr-Kni projection. (C) Hb-Gt-Kni projection. (D) Kr-Gt-Kni projection. The axes, xy-plane, and equilibria are as in Fig. S5. 10 trajectories are shown with starting points equally distributed on the Hb axis between 0–100. Time is represented as a color gradient along the trajectories, with start of cycle 13 as green, and gastrulation as red; trajectories are blue after gastrulation. The temporary reversals in trajectories are mitoses, during which the trajectories move toward the origin. (2.22 MB TIF) [file pcbi.1000303.s014.tif]

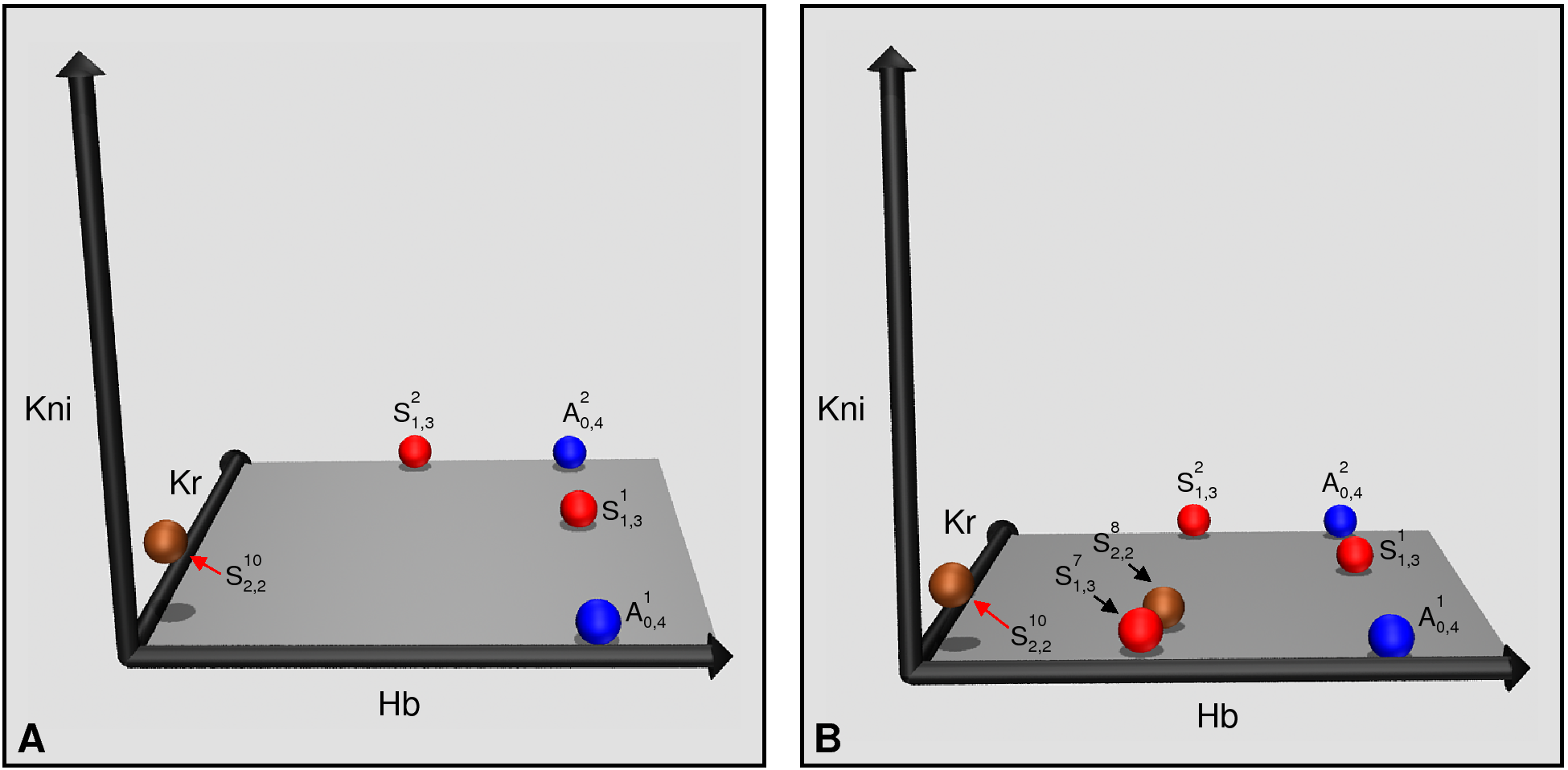

Supplement: Figure S7 — Bifurcations in the posterior region. Hb-Kr-Kni projection of phase portraits at (A) 53% EL and (B) 55% EL. The axes, xy-plane, and equilibria are as in Fig. S5. See Table S3 for bifurcation parameter values determined by the continuous analysis. Black arrows point to saddles, S71,3 and S82,2, that are created via a saddle-node bifurcation between 53% EL and 55% EL. (0.93 MB TIF) [file pcbi.1000303.s015.tif]

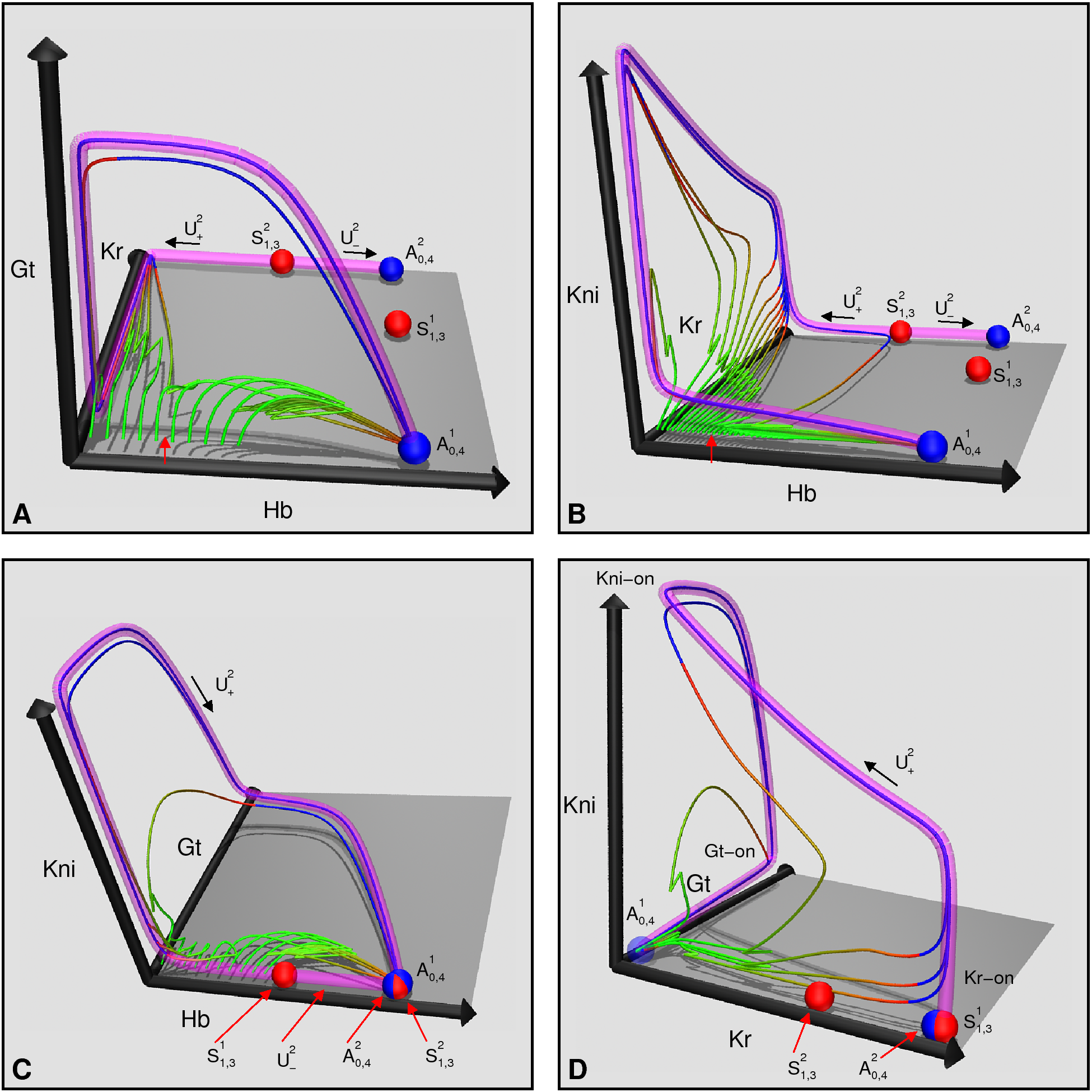

Supplement: Figure S8 — All four three-dimensional projections of the phase portrait at 57% EL. Axes, xy-plane, and equilibria are as in Fig. S5. All saddle equilibria are not shown (see Fig. S7). The unstable manifold of saddle S21,3, U2 is shown as a translucent magenta tube of radius 5. 10 trajectories are shown in panels A, C, and D, while 25 are shown in panel B. (A) Hb-Kr-Gt projection. Red arrow shows the separation of the indirect route trajectories from direct route ones. (B) Hb-Kr-Kni projection. Red arrow shows the separation of the indirect route trajectories from direct route ones. (C) Hb-Gt-Kni projection. (D) Kr-Gt-Kni projection. U2+ traverses the anteroposterior progression of gap gene states in the posterior region—Kr-on to kni-on to gt-on. (2.69 MB TIF) [file pcbi.1000303.s016.tif]

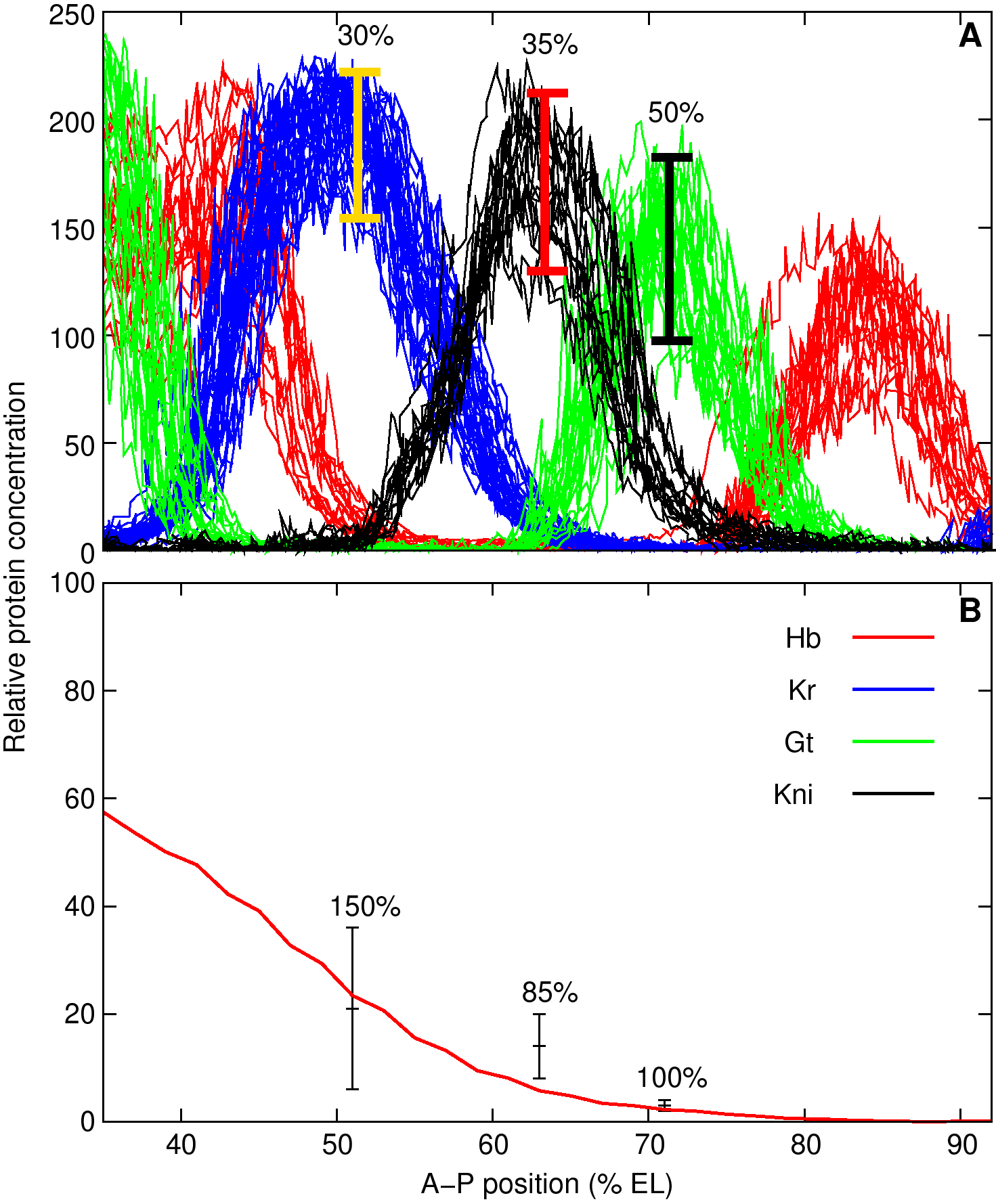

Supplement: Figure S9 — Tolerance to variation in maternal Hb. The range of initial conditions (B, error bars) for which modeled gap gene expression patterns have the same expression level variation as gap gene data in T8 (A). Error bars are ranges of concentrations, and percentage variation is the ratio of range to mean. (A) The variation in expression levels at the Kr peak is 30% (yellow bar), at the Kni peak is 35% (red bar), and at the Gt peak is 50% (black bar). (B) The tolerance range for maternal Hb is shown at three A–P positions (Kr, kni, and gt peaks). Maternal Hb profile is shown in red. The tolerance to initial variation is 150% at Kr peak, 85% at kni peak and 100% at gt peak. (0.46 MB TIF) [file pcbi.1000303.s017.tif]
