# Supplementary material for: Canalization of Gene Expression and Domain Shifts in the Drosophila Blastoderm by Dynamical Attractors
Source: PLoS Comput Biol. 2009 Mar 13;5(3):e1000303. doi: 10.1371/journal.pcbi.1000303 (PMC2646127; doi:10.1371/journal.pcbi.1000303)
Supplement: Table S1 — Time classes (0.02 MB PDF) [file pcbi.1000303.s018.pdf]

**Time Class**	**min from start of cycle 13** (t_i)	**min prior to gastrulation** $71.1 - t_i$
C13	10.550	60.550
T1	24.225	46.875
T2	30.475	40.625
T3	36.725	34.275
T4	42.975	28.125
T5	49.225	21.875
T6	55.475	15.625
T7	61.725	9.375
T8	67.975	3.125

Table S1: Time classes. The first column lists the nine time classes into which the expression data are classified. The second column lists the midpoint of each time class $t_i, i = 0, \ldots, 8$ at which the solution is calculated in the model. The last column lists the midpoints of time classes in minutes from gastrulation, which occurs 71.1 min after the onset of cleavage cycle 13 (based on [1]).

References

[1] Foe VE, Alberts BM (1983) Studies of nuclear and cytoplasmic behaviour during the five mitotic cycles that precede gastrulation in *Drosophila* embryogenesis. The Journal of Cell Science 61:31–70.
