# Supplementary material for: Canalization of Gene Expression and Domain Shifts in the Drosophila Blastoderm by Dynamical Attractors
Source: PLoS Comput Biol. 2009 Mar 13;5(3):e1000303. doi: 10.1371/journal.pcbi.1000303 (PMC2646127; doi:10.1371/journal.pcbi.1000303)
Supplement: Table S2 — Position of gap gene boundaries in the circuits with and without diffusion. (0.01 MB PDF) [file pcbi.1000303.s019.pdf]

| **Boundary** | **Position with diffusion (% EL)** | **Position without diffusion (% EL)** |
|---|---|---|
| *hb* posterior | 46 | 45–46 |
| *Kr* anterior | 43 | 45–46 |
| *Kr* posterior | 56 | 57–58 |
| *kni* anterior | 56 | 57–58 |
| *kni* posterior | 66 | 63–64 |
| *gt* anterior | 65 | 63–64 |

Table S2: Position of gap gene boundaries in the circuits with (first column) and without (second column) diffusion. The position of a domain boundary is the nucleus in which the concentration is half its maximum value in the domain and was calculated for model output in time class T8. For the diffusionless case, the domain boundaries are steep (see Fig. 2H). Hence, the pair of nuclei between which the concentration changes the most are shown in the second column.
