# Supplementary material for: Canalization of Gene Expression and Domain Shifts in the Drosophila Blastoderm by Dynamical Attractors
Source: PLoS Comput Biol. 2009 Mar 13;5(3):e1000303. doi: 10.1371/journal.pcbi.1000303 (PMC2646127; doi:10.1371/journal.pcbi.1000303)
Supplement: Table S3 — Comparison of bifurcation parameter values determined in the discrete analysis with the values determined in the continuous analysis. (0.02 MB PDF) [file pcbi.1000303.s020.pdf]

**Equilibria involved**	**Type**	**Newton-Raphson value (between %EL)**	**Continuous analysis value (at %EL)**
$S^3_{1,3}$–$S^4_{2,2}$	Annihilation	35–37	36.96
$S^5_{1,3}$–$S^6_{2,2}$	Creation	43–45	46.14
$A^3_{0,4}$–$S^5_{1,3}$	Annihilation	51–53	53.64
$S^7_{1,3}$–$S^8_{2,2}$	Creation	53–55	53.32
$S^9_{1,3}$–$S^6_{2,2}$	Annihilation	–	52.05
$S^9_{1,3}$–$S^{10}_{2,2}$	Creation	–	51.72

Table S3: Comparison of bifurcation parameter values determined in the discrete analysis with the values determined in the continuous analysis. All bifurcations are saddle-node bifurcations. The second column indicates whether the bifurcation involves a creation or annihilation of equilibria as the A–P position increases. The third column shows the nuclear positions between which the equilibria either appear or disappear in the discrete analysis. The last column shows the A–P positions for these bifurcations determined in the continuous analysis. The last two bifurcations are not observed in the discrete analysis as they occur between two consecutive analyzed nuclei and leave the number of equilibria and their type unchanged.
