# Supplementary material for: Canalization of Gene Expression and Domain Shifts in the Drosophila Blastoderm by Dynamical Attractors
Source: PLoS Comput Biol. 2009 Mar 13;5(3):e1000303. doi: 10.1371/journal.pcbi.1000303 (PMC2646127; doi:10.1371/journal.pcbi.1000303)
Supplement: Table S4 — Summary of all equilibria, the A–P region they exist in, and their function. (0.02 MB PDF) [file pcbi.1000303.s021.pdf]

Equilibrium	Existence (% EL)	Comments
$A^1_{0,4}$	35–71	*hb,gt*-on attractor until 41%, *hb*-on after 41%
$A^2_{0,4}$	35–71	*hb,Kr*-on attractor
$A^3_{0,4}$	35–53	*Kr*-on attractor, annihilated at 53.64% with $S^5_{1,3}$
$S^1_{1,3}$	35–71	
$S^2_{1,3}$	35–71	
$S^3_{1,3}$	35–37	Annihilated at 36.96% with $S^4_{2,2}$
$S^4_{2,2}$	35–37	Annihilated at 36.96% with $S^3_{1,3}$
$S^5_{1,3}$	46–54	Created at 46.14% with $S^6_{2,2}$ and annihilated at 53.64% with $A^3_{0,4}$
$S^6_{2,2}$	46–52	Created at 46.14% with $S^5_{1,3}$ and annihilated at 52.05% with $S^9_{1,3}$
$S^7_{1,3}$	53–71	Created at 53.32% with $S^8_{2,2}$
$S^8_{2,2}$	53–71	Created at 53.32% with $S^7_{1,3}$
$S^9_{1,3}$	51–52	Created at 51.72% with $S^{10}_{1,3}$ and annihilated at 52.05% with $S^6_{2,2}$
$S^{10}_{2,2}$	51–71	Created at 51.72% with $S^9_{1,3}$

Table S4: Summary of all equilibria, the A–P region they exist in, and their function. Creation and annihilation are with respect to motion from anterior to posterior. Bifurcation values were determined by the continuous analysis as described in Protocol S4.
